# Effects of structured exercise on executive function across childhood, adolescence, and later life: a systematic review and three-level meta-analysis examining exercise skill characteristics

**DOI:** 10.3389/fpsyg.2026.1952545

**Published:** 2026-10-14

**Authors:** Shuaichao Liu, Haiyan Fan, Shijie Zhou

**Affiliations:** 1 Institute of Physical Education and Training, Capital University of Physical Education and Sports, Beijing, China; 2 College of Plant Science and Technology, Beijing University of Agriculture, Beijing, China

**Keywords:** cognitive engagement, developmental stage, environmental predictability, executive function, motor skill learning, structured exercise, three-level meta-analysis

## Abstract

**Background:**

Structured exercise may benefit executive function across childhood, adolescence, and later life, but whether effects vary by executive-function domain, developmental stage, or qualitative exercise characteristics remains uncertain.

**Methods:**

We conducted a systematic review and three-level meta-analysis of randomized controlled trials in non-clinical children, adolescents, and older adults. Five databases were searched from inception to May 20, 2026; eligibility was restricted during screening to formally published English-language journal articles dated from January 1, 2015 to May 20, 2026. Outcomes were classified as inhibitory control, working memory, or cognitive flexibility. Intervention arms were coded for environmental predictability, cognitive engagement, coordination complexity, and motor skill learning demand. Three-level random-effects models retained dependent effect sizes within trials, with Study_ID-clustered CR2 robust inference and sensitivity analyses using alternative sampling-covariance structures. The review was registered in PROSPERO (CRD420261402803).

**Results:**

Forty-seven trials representing 49 independent samples and 4,195 actual participants contributed 202 effect-size estimates. Structured exercise was associated with a positive average effect on executive function (Hedges’ *g* = 0.412, robust 95% CI [0.284, 0.540], *p* < 0.001). Heterogeneity was substantial (total *I*^2^ = 74.45%), and the 95% prediction interval ranged from −0.476 to 1.299. Positive pooled estimates were observed for all three executive-function domains and for children, adolescents, and older adults, but neither domain (*p* = 0.060) nor developmental stage (*p* = 0.825) significantly moderated effects. Environmental predictability, cognitive engagement, coordination complexity, motor skill learning demand, intervention duration, and comparator type were also not significant moderators. Main conclusions were generally robust to alternative sampling-covariance assumptions and study restrictions, although stage-specific effect magnitudes, particularly in adolescents, were less stable in leave-one-trial-out analyses. Small-study-effect diagnostics were inconsistent, publication bias remained uncertain, and GRADE certainty was low because of serious risk of bias and inconsistency.

**Conclusion:**

Structured exercise was associated with better executive-function performance on average, but substantial heterogeneity and prediction intervals spanning no effect indicate that benefits are not uniform. Current evidence does not identify a specific executive-function domain, developmental stage, program duration, comparator type, or broadly defined exercise skill characteristic that reliably yields larger effects.

**Systematic review registration:**

https://www.crd.york.ac.uk/PROSPERO/view/CRD420261402803, identifier PROSPERO (CRD420261402803).

## 1 Introduction

Executive function refers to higher-order control processes supporting goal-directed behavior, adaptive decision-making, and flexible regulation of thought and action. Influential models distinguish three core components: inhibitory control, working memory, and cognitive flexibility or shifting (Miyake et al., 2000; Diamond, 2013; Friedman and Miyake, 2017). Together, these processes enable suppression of irrelevant responses, updating of task-relevant information, and switching between rules or mental sets, thereby supporting learning, self-regulation, health-related decision-making and independent functioning (Best et al., 2011; Moffitt et al., 2011; Hofmann et al., 2012; Diamond, 2013).

Intervention studies and meta-analyses suggest that physical activity and structured exercise can support cognitive function in children, adolescents, and older adults (Donnelly et al., 2016; Alvarez-Bueno et al., 2017; De Greeff et al., 2018; Northey et al., 2018; Chen et al., 2020). Proposed mechanisms include changes in arousal, cerebral perfusion, neurotrophic signaling, mood, and neural efficiency (Hillman et al., 2008; Erickson et al., 2011; Gomez-Pinilla and Hillman, 2013; Voss et al., 2013; Stillman et al., 2016). However, broader syntheses generally report small positive average effects with substantial heterogeneity across populations, exercise prescriptions, comparators, and cognitive outcomes (Ludyga et al., 2020; Zhang et al., 2023). Such variation may be important because executive-function domains differ in developmental trajectories and task demands (Miyake et al., 2000; Diamond, 2013; Karr et al., 2018; Ludyga et al., 2020).

Exercise interventions are commonly characterized by intensity, frequency, session duration, and program length, but these variables do not fully capture cognitive and motor demands. Attention has therefore shifted toward qualitative characteristics such as cognitive engagement, environmental predictability, coordination complexity, decision-making demand, novelty, motor learning, and adaptation to changing task constraints (Best, 2010; Pesce, 2012; Tomporowski et al., 2015; Diamond and Ling, 2016). Closed-skill activities are generally performed in stable environments, whereas open-skill activities require continuous adjustment to external information (Gu et al., 2019). Yet modality labels are only rough proxies for actual demands, which may vary with intervention design and delivery. Qualitative characteristics are therefore better judged from intervention content than modality name alone.

Cognitively engaging or coordinatively complex exercise may provide repeated opportunities to practice executive control in goal-directed contexts (Best, 2010; Pesce, 2012; Diamond and Ling, 2016). Some trials suggest larger executive-function benefits from cognitively engaging than less cognitively demanding aerobic exercise (Schmidt et al., 2015). Recent reviews in young populations also report favorable effects, although heterogeneous definitions, intervention designs, and possible small-study effects or publication bias limit interpretation (Mao et al., 2024; Teng et al., 2025). Whether distinct exercise skill characteristics explain variation in executive-function outcomes across randomized trials and developmental stages therefore remains unclear.

Developmental stage may also shape intervention effects. Executive function develops rapidly during childhood and adolescence alongside maturation of prefrontal and distributed neural systems (Best and Miller, 2010; Diamond, 2013), while exercise during these periods may act through physiological pathways and cognitively enriched movement experiences (Best, 2010; Tomporowski et al., 2015; Ludyga et al., 2020). In later life, age-related changes in brain structure and neural efficiency may increase the relevance of interventions supporting cardiovascular, metabolic, and functional health (Park and Reuter-Lorenz, 2009; Salthouse, 2010; Erickson et al., 2011; Northey et al., 2018). Nevertheless, meta-analyses have not produced consistent evidence that effects vary by age, exercise modality, dose, or cognitive domain (Chen et al., 2020; Ludyga et al., 2020; Zhang et al., 2023).

Several gaps remain. Most reviews have focused on a single developmental period or broad cognitive outcomes, and few have examined the three core executive-function domains together with multiple qualitative intervention characteristics (Ludyga et al., 2020; Zhang et al., 2023). Reviews of open- versus closed-skill exercise or cognitively engaging physical activity have often included observational evidence, concentrated on younger populations, or treated cognitive engagement as a single broad construct (Gu et al., 2019; Mao et al., 2024; Teng et al., 2025). It therefore remains uncertain whether these qualitative characteristics account for distinct sources of heterogeneity across non-clinical randomized controlled trials.

A further analytical challenge is that exercise–cognition trials often report multiple eligible tasks, outcomes, domains, or intervention contrasts. Selecting one result per trial may discard information, whereas averaging results can obscure domain- or task-specific variation. Three-level meta-analysis allows dependent effect sizes to be retained while modeling their nesting within trials (Van den Noortgate et al., 2013; Cheung, 2014; Assink and Wibbelink, 2016). Accordingly, this review examined structured exercise effects on inhibitory control, working memory, and cognitive flexibility in children, adolescents, and older adults, and tested whether developmental stage, environmental predictability, cognitive engagement, coordination complexity, motor skill learning demand, intervention duration, and comparator type moderated effect size.

## 2 Methods

### 2.1 Protocol, registration, and reporting

This systematic review and meta-analysis was registered in PROSPERO (CRD420261402803) on May 22, 2026. Versions 2.0 and 3.0 were published on July 14 and July 26, 2026, respectively. Because both updates followed the initial synthesis, post-registration decisions were treated as retrospective; analyses added or materially revised thereafter were classified as exploratory or *post hoc*, as appropriate. Changes across versions, including unimplemented plans and methodological refinements, are documented with their timing and rationale in Supplementary Table S5. No separate review protocol was prepared or published beyond the PROSPERO registration record. The review followed PRISMA 2020 and its explanation and elaboration guidance (Page et al., 2021a, 2021b) and was informed by the *Cochrane Handbook for Systematic Reviews of Interventions*, Version 6.5 (Higgins et al., 2024). As only aggregate published data were used, ethics approval and informed consent were not required.

### 2.2 Eligibility criteria

Eligibility was defined using the PICOS framework (Higgins et al., 2024; Page et al., 2021a). Eligible participants were non-clinical children, adolescents, or older adults. Children were defined as samples with a mean age <12 years, adolescents as 12 to <18 years, and older adults as samples with a mean age or reported lower age limit ≥60 years. When age ranges crossed developmental boundaries, classification was based primarily on mean age, supported by the population description and recruitment setting. Mixed middle-aged and older samples were included only when the mean age was ≥60 years and the sample was described as older or aging. Samples with diagnosed neurological, psychiatric, neurodevelopmental, or cognitive disorders were excluded, whereas obesity, physical inactivity, low fitness, socioeconomic disadvantage, and other non-neurological characteristics did not themselves determine ineligibility.

Structured exercise was defined as a planned and organized program of purposeful bodily movement delivered according to a prespecified protocol (Caspersen et al., 1985). Eligible interventions included aerobic, resistance, multicomponent, coordinative, mind–body, dance, exergaming, sport-based, physically active academic, and motor–cognitive or dual-task programs. Interventions consisting solely of cognitive training or other nonexercise therapies were excluded.

Eligible comparators included no intervention, wait-list, usual activity or care, routine physical education or physical activity not prescribed as an additional intervention, and active nonexercise controls. Studies were excluded when all comparator arms received investigator-prescribed structured exercise; in multi-arm trials, each eligible exercise arm was compared with the relevant eligible comparator.

Eligible outcomes were performance-based measures of inhibitory control, working memory, or cognitive flexibility (Miyake et al., 2000; Diamond, 2013; Friedman and Miyake, 2017). Global or unassignable cognitive measures and non-executive outcomes were excluded.

Eligible designs were randomized controlled trials with a concurrent comparator, including parallel-group, cluster-randomized, multi-arm, and crossover trials. Non-randomized or uncontrolled designs and non-original research reports were excluded. Quantitative synthesis additionally required sufficient information to calculate at least one standardized effect size from reported summary statistics or convertible test results.

### 2.3 Search strategy and study selection

PubMed, Embase, the Cochrane Library, Scopus, and Web of Science were searched from inception to May 20, 2026, without date or language filters in the database interfaces. During screening, eligibility was restricted to formally published English-language journal articles dated January 1, 2015 to May 20, 2026. The 2015 cutoff was a pragmatic scope restriction intended to focus on approximately the most recent decade of contemporary randomized evidence relevant to current intervention designs, executive-function assessment practices, and qualitative exercise characteristics; it did not imply that earlier trials were methodologically inferior or irrelevant. The review should therefore be interpreted as contemporary rather than historically exhaustive.

Dissertations, conference proceedings, trial registries, and other grey-literature sources were not searched, and neither backward reference-list nor forward citation searching was undertaken. These language, publication-status, and supplementary-identification restrictions may have reduced evidence completeness and representativeness and are addressed in the limitations. The 2015 cutoff and the English-language and journal-publication restrictions were introduced after the original PROSPERO registration, were based on bibliographic characteristics rather than study findings, and are documented in Supplementary Table S5.

Search strategies combined controlled vocabulary, where available, with free-text terms for exercise or physical activity, executive function, developmental stage, and randomized study design, including the three executive-function domains, common exercise modalities, and relevant cognitive-control tasks. Syntax was adapted to each database. Full strategies, field codes, exclusions, search dates, and yields are provided in Supplementary Table S1. Search strategies were internally checked against relevant elements of the PRESS 2015 guideline (McGowan et al., 2016; Rethlefsen et al., 2021).

Records were imported into EndNote X9 and deduplicated electronically with manual verification. Two reviewers (SCL and HYF) independently screened titles/abstracts and then full texts; disagreements were resolved by discussion or, when necessary, a third reviewer (SJZ) (Higgins et al., 2024). No automated screening or machine-learning prioritization was used. Full-text exclusion reasons were recorded by category, including insufficient quantitative data for effect-size calculation. Reports describing the same or overlapping participant samples were linked and treated as a single study; information was combined using the most complete source for each data item, with duplicate participants and outcomes counted only once. Study selection is summarized in the PRISMA 2020 flow diagram (Page et al., 2021a).

### 2.4 Data extraction and outcome classification

Data were extracted using a standardized, pilot-tested form informed by systematic-review guidance (Higgins et al., 2024; Page et al., 2021a). Two reviewers (SCL and HYF) independently completed extraction, with disagreements resolved through discussion or consultation with a third reviewer (SJZ).

Extracted information covered study design and publication details, participant characteristics, developmental stage, sample size, intervention and comparator characteristics, and outcome-level data required for effect-size calculation. Intervention reporting was informed by TIDieR and CERT principles (Hoffmann et al., 2014; Slade et al., 2016) and collected in sufficient detail to support the skill-characteristic coding described in Section 2.5. For each eligible outcome, we recorded the task, assessment condition, performance metric, time point, scoring direction, analyzed sample size, and relevant summary statistics. Data were obtained from published articles and Supplementary materials; study authors were not contacted, as documented in Supplementary Table S5.

Executive-function outcomes were classified as inhibitory control, working memory, or cognitive flexibility (Miyake et al., 2000; Diamond, 2013; Friedman and Miyake, 2017). Because executive-function tasks are not process-pure, classification was made at the outcome level using task procedures, the construct identified by the study authors, the specific condition, and the extracted metric rather than the task name alone. Inhibitory-control outcomes primarily reflected suppression of prepotent or conflicting responses, working-memory outcomes maintenance or updating of information, and cognitive-flexibility outcomes shifting between rules or task sets.

Distinct eligible indices from the same task were retained as separate effect sizes, and scoring direction was recorded for harmonization as described in Section 2.7. Developmental stage was assigned at the independent-sample level using Section 2.2 criteria; subgroups were treated separately only when participant and outcome data were independently extractable.

### 2.5 Coding of exercise skill characteristics

Exercise skill characteristics were coded at the intervention-arm level using a review-specific framework informed by theories of cognitively engaging and skill-based physical activity (Best, 2010; Pesce, 2012; Tomporowski et al., 2015; Diamond and Ling, 2016). All effect sizes from the same intervention arm received the same codes. Classification reflected the program’s central and recurring demands rather than modality labels or isolated activities.

Environmental predictability was classified as stable, semi-dynamic, or dynamic. Stable programs involved largely predictable or self-paced movement; semi-dynamic programs followed relatively stable rules but required periodic responses to changing cues or task conditions; dynamic programs required frequent adaptation to changing stimuli, partners, opponents, objects, or movement conditions. This framework extended the conventional open- versus closed-skill distinction (Gu et al., 2019).

Cognitive engagement, coordination complexity, and motor skill learning demand were initially coded as low, moderate, or high; because low and moderate categories were sparse, they were combined *post hoc* as low-to-moderate for meta-regression. High cognitive engagement involved recurring demands for monitoring, response selection, inhibition, updating, switching, decision-making, or adaptation. High coordination complexity involved substantial multisegmental or bilateral coordination, dynamic balance, spatial–temporal precision, sequential organization, or object manipulation. Motor skill learning demand was high when acquiring, refining, progressing, or adapting novel or technically complex skills was central to the intervention. Broad skill classification was recorded as closed-, mixed-, or open-skill, and skill progression as present, absent, or unclear.

Two reviewers (SCL and HYF) independently coded each intervention arm using the main report and available Supplementary materials, protocols, or trial registrations. Disagreements were resolved by consensus or, when necessary, consultation with a third reviewer (SJZ). The reviewer-specific pre-consensus coding files were not retained; therefore, inter-rater agreement statistics such as Cohen’s kappa could not be retrospectively calculated without a new coding exercise, and no retrospective reliability coefficient was generated. Coding confidence was rated as high or moderate according to intervention-reporting completeness and clarity.

During revision, the operational definitions and decision rules were consolidated in a revision-stage coding codebook to improve transparency and reproducibility. The codebook documents interpretation of the final consensus codes but does not represent a new independent recoding exercise or retrospective reliability assessment. Final arm-level codes and rationales are reported in Supplementary Table S2, with operational definitions and decision rules provided in Supplementary Dataset S1.

### 2.6 Risk of bias assessment

Risk of bias was assessed using the revised Cochrane Risk of Bias tool for randomized trials (RoB 2), with the target estimand defined as the effect of assignment to intervention on eligible executive-function outcomes (Sterne et al., 2019). Standard and design-specific RoB 2 versions were used for individually randomized, cluster-randomized, and crossover trials as appropriate.

Assessments covered the randomization process, deviations from intended interventions, missing outcome data, outcome measurement, and selection of the reported result. Domain-level and overall judgments were classified as low risk, some concerns, or high risk according to RoB 2 algorithms.

Two reviewers (SCL and HYF) independently completed assessments using available reports and supporting materials, with disagreements resolved by discussion or consultation with a third reviewer (SJZ). When judgments differed across eligible outcomes within a trial, the most severe overall judgment was used for trial-level summaries and the high-risk-trial sensitivity analysis. Results were displayed using traffic-light and weighted summary plots (McGuinness and Higgins, 2021).

### 2.7 Effect-size calculation and dependency handling

The primary effect-size measure was Hedges’ *g*. For independent parallel-group contrasts, post-intervention standardized mean differences were calculated using the pooled post-intervention standard deviation:


$$S_{p}=\sqrt{\frac{(n_{E}-1)S_{E}^{2}+(n_{C}-1)S_{C}^{2}}{n_{E}+n_{C}-2}},$$

and


$$d=\frac{M_{E}-M_{C}}{S_{p}},$$

where the terms denote post-intervention means, standard deviations, and analyzed sample sizes in the structured-exercise and comparator groups. Cohen’s *d* was corrected for small-sample bias to obtain Hedges’ *g* (Hedges, 1981), with sampling variance calculated from the same group-level information.

Effect directions were harmonized so that positive values favored structured exercise. Outcomes for which lower values indicated better performance, including reaction time, completion time, error rates, interference costs, and switch costs, were reverse-coded. When post-intervention means and standard deviations were unavailable, eligible between-group test statistics and group sizes were used to derive Hedges’ *g*. For one outcome, the reported control-group post-intervention standard deviation was inconsistent with the reported *t* statistic and Cohen’s *d*; the effect size was therefore derived from the *t* statistic and group sizes rather than by imputing or modifying the inconsistent standard deviation. Derivations were checked against source reports and documented in the analysis records.

Each trial, independent participant sample, intervention arm, comparator arm, and effect-size estimate received a unique identifier. Multiple eligible outcomes and contrasts were retained rather than averaged. Shared intervention or comparator arms were linked across relevant contrasts and not treated as independent participant groups. These identifiers supported the dependency-aware analyses described in Section 2.8.

Actual participant counts used for reporting were distinguished from sample sizes used for effect-size variance calculations. For cluster-randomized trials requiring adjustment, design-adjusted effective sample sizes were used for variance estimation, whereas actual analyzed participant numbers were retained for participant reporting; shared participants were not multiply counted.

For cluster-randomized trials without usable cluster-adjusted estimates, effective sample sizes were calculated using the design effect: DE=1+(m−1)ICC , where the terms represent mean cluster size and the intracluster correlation coefficient (ICC) (Higgins et al., 2024). Group means and standard deviations were unchanged. Trial-specific behavioral-outcome ICCs were used when available; otherwise, ICC = 0.05 was used for the primary adjustment, with alternative ICCs examined in sensitivity analyses.

For crossover and other repeated-condition trials, effect sizes were derived from paired contrasts, paired test statistics, or appropriately adjusted model estimates. Separate condition means were not treated as independent parallel groups unless independently analyzable first-period data were available. Morris pre–post change estimates were calculated for sensitivity analyses (Morris, 2008). Design-specific derivations and adjustments were retained in the analysis logs.

### 2.8 Statistical analysis

All statistical analyses were conducted in R version 4.6.1. Multilevel meta-analysis and sampling variance–covariance matrices used metafor version 5.0–1, and cluster-robust inference used clubSandwich version 0.7.0.

#### 2.8.1 Primary model, robust inference, and heterogeneity

The primary analysis used an intercept-only three-level random-effects model fitted with metafor::rma.mv(), retaining all eligible effect sizes nested within trials (Van den Noortgate et al., 2013; Cheung, 2014; Assink and Wibbelink, 2016). Level 1 represented sampling error, Level 2 residual heterogeneity among effect sizes within trials, and Level 3 heterogeneity between trials. The random-effects structure was ~1 | Study_ID / Effect_ID, with variance components estimated by restricted maximum likelihood (REML). Independent_sample_ID identified independent participant samples for data management and sampling-covariance sensitivity analyses but was not modeled as an additional random-effects level.

The primary working sampling variance matrix was diagonal: estimated sampling variances were placed on the diagonal and off-diagonal covariances set to zero. This did not assume complete independence of retained effects, because dependence was additionally addressed through the nested random-effects structure and Study_ID-clustered robust inference; the zero-covariance assumption was relaxed in sensitivity analyses using non-diagonal variance–covariance matrices.

The intercept represented pooled Hedges’ *g*, with positive values favoring structured exercise. Coefficient-level inference used cluster-robust variance estimation with the CR2 small-sample correction, clustered by Study_ID (Tipton, 2015; Pustejovsky and Tipton, 2018). Robust standard errors, hypothesis tests, and 95% confidence intervals used Satterthwaite-adjusted degrees of freedom and the CR2 covariance estimator in clubSandwich. CR2 affected coefficient-level uncertainty but not fitted coefficients or REML variance-component estimates. Tests were two-sided with α = 0.05.

Heterogeneity was summarized by the between-trial and residual within-trial effect-size variance components, together with study-level, effect-size-level, and total multilevel *I*^2^ relative to the typical sampling variance. Because *I*^2^ depends partly on the sampling-variance structure, interpretation considered both absolute variance components and *I*^2^.

The 95% prediction interval was obtained with metafor::predict(), incorporating both heterogeneity components and not the CR2 covariance estimator. It was interpreted as the approximate range of true effects for a future eligible intervention–comparator contrast in a comparable setting (Higgins et al., 2009; IntHout et al., 2016).

#### 2.8.2 Moderator analyses

Executive-function domain, developmental stage, exercise skill characteristics, intervention duration, and comparator type were examined in separate three-level meta-regression models retaining the primary random-effects structure, REML estimation, and Study_ID-clustered CR2 inference unless otherwise stated. Category-specific pooled effects used no-intercept models, whereas between-category differences used reference-coded models. Reference categories were inhibitory control, childhood, stable environmental predictability, and low-to-moderate demand for binary skill moderators. Moderators with more than two categories were evaluated using robust Wald-type omnibus *F* tests with the Hotelling-type small-sample correction (HTZ) (Tipton, 2015; Pustejovsky and Tipton, 2018). Moderation was judged from direct contrasts or omnibus tests rather than differences in significance across category-specific estimates.

Executive-function domain comprised inhibitory control, working memory, and cognitive flexibility; developmental stage comprised childhood, adolescence, and later life. These moderators were analyzed separately without mutual adjustment. The domain-by-stage interaction specified in PROSPERO Version 2.0 was not fitted because cross-classified cells contained too few independent trials for stable CR2 inference; this departure is documented in Supplementary Table S5.

Environmental predictability was analyzed as stable, semi-dynamic, or dynamic, whereas cognitive engagement, coordination complexity, and motor skill learning demand were analyzed as high versus low-to-moderate. The low and moderate categories were combined *post hoc* as described in Section 2.5. Broad skill classification and skill progression were summarized descriptively but not meta-regressed because open-skill and no-progression categories were too sparse. The four skill dimensions were modeled separately because of conceptual and empirical overlap and limited trial information.

Intervention duration was examined continuously. A linear model centered duration at 12 weeks and expressed coefficients per additional four weeks; a second model used log₂-transformed duration, representing a doubling of program length. Because only two trials exceeded 16 weeks, a *post hoc* analysis was restricted to 2–16 weeks.

Comparator conditions were classified as passive/usual-activity/usual-care, active nonexercise attention/low-demand, or usual physical education/routine physical activity. Environmental predictability and cognitive engagement were additionally examined in separate models adjusted for executive-function domain, developmental stage, and comparator type. Coordination complexity and motor skill learning demand were not adjusted because of overlap and overparameterization risk.

Moderator coefficients were interpreted as aggregate-level observational associations rather than independent causal effects (Thompson and Higgins, 2002). No multiplicity adjustment was applied across exploratory analyses; *p* values were interpreted cautiously. Analytical status and timing are reported in Supplementary Table S5.

#### 2.8.3 Sensitivity analyses and assessment of small-study effects

Sensitivity analyses examined robustness to assumptions about sampling dependence, cluster adjustment, effect-size construction, baseline imbalance, risk of bias, intervention coding, comparator selection, and trial influence. Unless otherwise stated, analyses retained the primary three-level random-effects structure, REML estimation, and Study_ID-clustered CR2 inference; timing and registration status are reported in Supplementary Table S5.

The primary diagonal sampling-variance matrix was compared with non-diagonal matrices constructed using metafor::vcalc(). Covariance blocks were defined by Study_ID and Independent_sample_ID; Effect_ID identified estimates, and Intervention_Arm_ID and Control_Arm_ID identified shared arms. N_E_variance and N_C_variance were used as arm-size inputs, including design-adjusted effective sample sizes where applicable, and were kept distinct from actual participant counts. Within-sample correlations of ρ = 0.30, 0.50, and 0.70 were examined. The vcalc() structure represented shared-arm overlap and repeated outcomes within the same independent sample, with zero covariance assigned to non-overlapping samples. For each ρ, the overall model was refitted without changing its fixed- or random-effects specification, and the pooled effect, CR2 inference, heterogeneity variance components, multilevel *I*^2^, and prediction interval were re-evaluated.

Sampling-covariance sensitivity was also examined for six moderator models: executive-function domain, developmental stage, environmental predictability, cognitive engagement, coordination complexity, and motor skill learning demand. Coding, contrasts or omnibus tests, random-effects structure, and CR2 inference were unchanged; only the sampling variance–covariance matrix varied across conditions. Developmental-stage robustness was additionally assessed using separate intercept-only three-level models for childhood, adolescence, and later life under the same covariance structures. These models estimated heterogeneity separately within each stage, were distinct from the developmental-stage meta-regression in Section 2.8.2, and retained Study_ID-clustered CR2 inference with Satterthwaite-adjusted degrees of freedom.

Additional sensitivity analyses recalculated cluster-adjusted effective sample sizes, effect sizes, and variances using ICCs of 0.01, 0.05, and 0.10 and excluded the trial requiring an assumed ICC. Post-intervention Hedges’ *g* estimates were compared with Morris change-score estimates assuming pre–post correlations of 0.30, 0.50, and 0.70 (Morris, 2008). Further analyses excluded estimates with absolute baseline standardized mean differences ≥0.80 or ≥0.50, high-risk trials, or comparator conditions other than passive, no intervention, wait-list, usual activity, or usual care. Primary and skill-characteristic models were also restricted to high-confidence coded arms.

Trial influence was assessed by leave-one-trial-out analyses removing one complete Study_ID at each iteration, both overall and separately within childhood, adolescence, and later life under the primary diagonal structure. Robustness was evaluated from changes in pooled estimates and CR2 confidence intervals rather than isolated changes in statistical significance. A protocol-planned exclusion based solely on an extreme estimate was not retained after source data and calculations were verified; influence was instead evaluated through these analyses.

Potential small-study effects were assessed using dependency-aware multilevel models and complementary study-level diagnostics. Funnel-plot asymmetry was not treated as direct evidence of publication bias because it may reflect heterogeneity, methodological differences, or sampling variation (Sterne et al., 2011). The primary multilevel diagnostic used


$$x_{i}=\sqrt{\frac{1}{n_{\mathrm{Ei}}^{*}}+\frac{1}{n_{\mathrm{Ci}}^{*}}}$$

as a moderator, where the terms represented design-adjusted effective sample sizes of the exercise and comparator arms; design-appropriate participant information was used for repeated-condition contrasts (Pustejovsky and Rodgers, 2019). Models were fitted under the primary diagonal structure and a non-diagonal matrix with ρ = 0.50. An Egger-type multilevel model using the sampling standard error of Hedges’ *g* was also fitted under both structures and interpreted cautiously because of possible mathematical coupling (Egger et al., 1997; Pustejovsky and Rodgers, 2019).

For study-level diagnostics, estimates were combined within trials by generalized least squares using study-specific covariance matrices with ρ = 0.50. The resulting 47 trial-level estimates were examined using classical Egger regression, the Begg–Mazumdar rank-correlation test, and a contour-enhanced funnel plot (Begg and Mazumdar, 1994; Egger et al., 1997; Peters et al., 2008). An auxiliary REML random-effects model summarized the aggregated estimates but did not replace the primary analysis. These procedures were *post hoc*, and no conventional asymmetry test was applied directly to all 202 dependent estimates. Conclusions were based on consistency across diagnostics rather than any single test.

### 2.9 Certainty of evidence

Certainty of evidence was evaluated using GRADE for overall executive-function performance and separately for inhibitory control, working memory, and cognitive flexibility (Balshem et al., 2011; Guyatt et al., 2011). The overall outcome summarized the three domains and was not statistically independent of the domain-specific evidence. Randomized evidence started at high certainty and could be downgraded for risk of bias, inconsistency, indirectness, imprecision, or publication bias.

Risk of bias was informed by RoB 2 assessments and sensitivity analyses; inconsistency by effect dispersion, variance components, multilevel *I*^2^, prediction intervals, and moderator findings; indirectness by the review question; and imprecision by robust confidence intervals and numbers of independent trials and participants. Because no validated minimally important difference applied across these measures, this limitation was considered explicitly. Publication bias was evaluated from search coverage, study-size distribution, and multilevel and study-level diagnostics.

Two reviewers (SCL and HYF) independently rated certainty as high, moderate, low, or very low, resolving disagreements by discussion or third-reviewer consultation (SJZ). Detailed judgments are reported in Supplementary Table S6.

## 3 Results

### 3.1 Study selection

The searches identified 18,346 records. After removal of 7,849 duplicates, 10,497 records underwent title/abstract screening, of which 10,359 were excluded. All 138 reports sought for retrieval were obtained and assessed for eligibility. Full-text assessment excluded 91 reports because of insufficient quantitative data (*n* = 36), an ineligible comparator (*n* = 28), executive-function outcome (*n* = 9), study design (*n* = 7), intervention (*n* = 4), publication type (*n* = 4), or duplicate/secondary publication (*n* = 3). Ultimately, 47 reports representing 47 randomized controlled trials were included in both the systematic review and meta-analysis (Figure 1).

**Figure 1:**
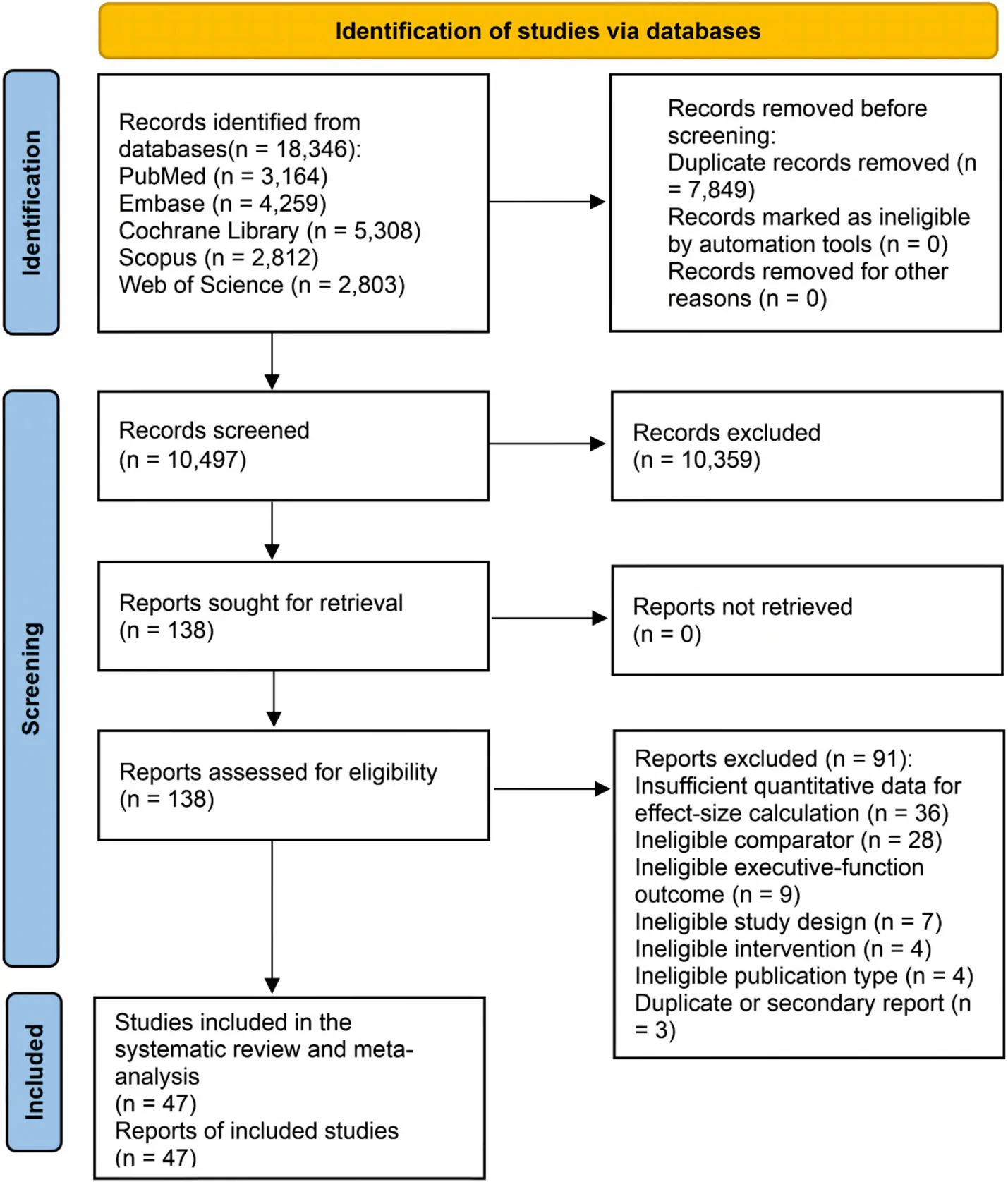
PRISMA 2020 flow diagram of the identification, screening, eligibility assessment, and inclusion of randomized controlled trials examining structured exercise and executive function.

### 3.2 Study and effect-size characteristics

The quantitative synthesis included 47 randomized controlled trials comprising 49 independent participant samples, 61 structured-exercise arms, 49 comparator arms, and 202 effect-size estimates. In total, 4,195 actual participants were analyzed, including 2,199 in structured-exercise arms and 1,996 in comparator arms. These counts were distinct from design-adjusted effective sample sizes used for effect-size variance or sampling-covariance calculations. Trials were published between 2015 and 2026, and interventions lasted 2–44 weeks.

Seventeen trials of children contributed 18 independent samples, 90 effect sizes, and 2,250 participants; nine adolescent trials contributed 10 samples, 30 effects, and 756 participants; and 21 trials of older adults contributed 21 samples, 82 effects, and 1,189 participants. The adolescent evidence base was therefore smaller than those for children and older adults.

Inhibitory control comprised 89 effect sizes from 31 trials and 32 samples involving 3,183 participants; working memory, 51 effects from 22 trials and 24 samples involving 2,703 participants; and cognitive flexibility, 62 effects from 27 trials and 28 samples involving 2,035 participants. Because trials and samples could contribute to multiple domains, these participant counts overlap and should not be summed.

Interventions spanned aerobic, resistance, coordinative, mind–body, dance, exergaming, sport-based, physically active academic, and motor–cognitive programs. Comparator conditions comprised passive/usual-activity/usual-care controls for 116 effects from 29 trials, active nonexercise attention/low-demand controls for 30 effects from nine trials, and usual physical education/routine physical activity for 56 effects from 10 trials; trial counts could overlap across comparator categories.

Environmental predictability was coded as stable for 39 arms, semi-dynamic for 13, and dynamic for nine. Cognitive engagement was high for 33 arms and low-to-moderate for 28; coordination complexity was high for 31 and low-to-moderate for 30; and motor skill learning demand was high for 27 and low-to-moderate for 34. Coding confidence was high for 57 arms and moderate for four. Only two open-skill arms and two arms without skill progression were identified, so these variables were summarized descriptively rather than meta-regressed. Trial characteristics and arm-specific sample sizes are reported in Table 1, and arm-level coding in Supplementary Table S2.

**Table 1:** Study characteristics and arm-specific sample sizes of the 47 randomized controlled trials included in the primary three-level meta-analysis.

Study	Country	Developmental stage	Structured-exercise intervention arm(s), *n* randomized/Analyzed	Comparator arm(s), *n* randomized/Analyzed	Duration	EF domain(s)
Alexandrino et al. (2026)	Brazil	Adolescence	Short-duration HIIT group (*n* = 62/46)	Regular physical education control (*n* = 138/115)	6 weeks	IC; CF
Antunes et al. (2015)	Brazil	Later life	Aerobic exercise training group (*n* = 22/22)	No-exercise control group (*n* = 23/23)	26 weeks	WM; CF
Basarir et al. (2025)	Türkiye	Childhood	Coordination-based training group / Exercise group (EG) (*n* = 26/26)	Control group (CG) (*n* = 25/25)	8 weeks	IC
Cai et al. (2025)	China	Adolescence	Classroom physical education exercise intervention group (*n* = 114/114)	Control group / normal school program (*n* = 108/108)	12 weeks	WM
Chen et al. (2016)	Taiwan	Adolescence	Moderate-intensity physical activity program (*n* = 31/25)	Wait-list control / regular health education (*n* = 29/25)	12 weeks	CF
Cheng et al. (2023)	China	Childhood	Equine-assisted activity group (EAAG) (*n* = 12/12)	Control group (CG) (*n* = 12/12)	12 weeks	IC; WM; CF
Cho et al. (2017)	South Korea	Childhood	Taekwondo training (TKD) (*n* = 15/15)	Control (*n* = 15/15)	16 weeks	IC
Coetsee and Terblanche (2017)	South Africa	Later life	High-intensity aerobic interval training (HIIT) (*n* = 13/11) Moderate continuous aerobic training (MCT) (*n* = 13/13) Resistance training (RT) (*n* = 24/22)	Non-exercise control (CON) (*n* = 22/19)	16 weeks	IC
Cunha et al. (2026)	Brazil	Later life	8–12 RM resistance training (*n* = 50/50) 10–15 RM resistance training (*n* = 50/50)	Non-exercise control group (*n* = 50/50)	12 weeks	IC; CF
de Greeff et al. (2016)	Netherlands	Childhood	Physically active academic lessons (F&V) (*n* = 249/176)	Regular lessons / usual classroom curriculum (*n* = 250/167)	44 weeks	IC; WM; CF
García-Garro et al. (2020)	Spain	Later life	Pilates group (*n* = 55/55)	Control group (*n* = 55/52)	12 weeks	CF
Goumopoulos et al. (2026)	Greece	Later life	GAME2AWE exergaming intervention (*n* = 30/30)	Usual care control (*n* = 30/28)	16 weeks	WM; CF
Hola et al. (2024)	Czech Republic	Later life	Dance group (DG) (*n* = 25/20) Martial arts group (MaG) (*n* = 25/16)	Control group (CG) (*n* = 27/23)	12 weeks	CF
Intawachirarat et al. (2026)	Thailand	Childhood	Dance group (*n* = 18/17)	Control group (*n* = 18/18)	10 weeks	IC; WM; CF
Iuliano et al. (2015)	Italy	Later life	Cardiovascular Group (CVG) (*n* = 20/20) Resistance Group (RG) (*n* = 20/20) Postural Group (PG) (*n* = 20/20)	No-training control group (CG) (*n* = 20/20)	12 weeks	IC; CF
Kaseb et al. (2026)	Iran	Later life	Resistance training (RT) (*n* = 16/16) Resistance training with blood flow restriction (RT-BFR) (*n* = 19/19)	Control group (*n* = 16/16)	8 weeks	CF
Kim and So (2015)	South Korea	Childhood	Combined Exercise Training (CET) (*n* = 11/10)	No-exercise control (*n* = 11/10)	16 weeks	IC
Ladawan et al. (2022)	Thailand	Later life	4-movement Qigong exercise (*n* = 13/13)	Usual activities of daily living control (*n* = 13/13)	8 weeks	CF
Liu et al. (2018)	Taiwan	Adolescence	Coordination exercise / jump-rope training (*n* = 40/35)	Wait-list control (*n* = 40/35)	12 weeks	IC
Liu W. et al. (2025)	China	Childhood	DanceSport intervention, 4–5 years (*n* = 48/44) DanceSport intervention, 5–6 years (*n* = 59/53)	Regular kindergarten activities, 4–5 years (*n* = 48/46) Regular kindergarten activities, 5–6 years (*n* = 59/58)	8 weeks	IC; WM; CF
Liu Y. et al. (2025)	China	Childhood	High-intensity interval rope skipping (HIIT) (*n* = 20/18) Moderate-intensity continuous rope skipping (MICT) (*n* = 20/19)	Regular activities control group (*n* = 20/19)	8 weeks	IC
Lu et al. (2016)	Hong Kong, China	Later life	12-form Yang-style Tai Chi training (*n* = 15/15)	General interest classes / social control (*n* = 16/16)	16 weeks	IC
Ludyga et al. (2018)	Switzerland	Adolescence	Combined aerobic and coordinative school-break exercise (*n* = 20/17)	Wait-list / social-interaction control (*n* = 16/16)	8 weeks	WM
Ludyga et al. (2019)	Germany	Childhood	Aerobic training (*n* = 15/11) Coordinative training (*n* = 15/12)	Assisted homework sessions (*n* = 15/14)	10 weeks	IC
Martín-Martínez et al. (2015)	Spain	Adolescence	Additional small-sided games physical activity program (*n* = 27/27)	Ordinary school physical education control (*n* = 27/27)	8 weeks	IC; CF
Meijer et al. (2021)	Netherlands	Childhood	Aerobic exercise intervention (*n* = 221/206) Cognitively demanding exercise intervention (*n* = 240/235)	Regular physical education control (*n* = 430/415)	14 weeks	IC; WM
Milot et al. (2026)	France	Later life	Physical training (PT) group (*n* = 12/9)	Active control (AC): health education (*n* = 11/10) Passive control (PC): no intervention (*n* = 11/11)	12 weeks	IC; CF
Moghadam et al. (2025)	Iran	Later life	Body percussion rhythmic exercise group (*n* = 30/30)	Routine daily activity control group (*n* = 30/30)	12 weeks	CF
Moreau et al. (2017)	New Zealand	Childhood	High-intensity training (HIT) (*n* = 159/152)	Active placebo/non-exercise control (*n* = 159/153)	6 weeks	IC; WM
Nishiguchi et al. (2015)	Japan	Later life	Physical and cognitive exercise program (*n* = 24/24)	No-intervention usual-time control (*n* = 24/24)	12 weeks	WM; CF
Pacheco Lopes Filho et al. (2019)	Brazil	Later life	Karate Group (*n* = 31/16)	Control Group (*n* = 31/17)	12 weeks	CF
Park (2022)	South Korea	Later life	Moderate-intensity aerobic stepping exercise training (*n* = 28/28)	Wait-list control group (*n* = 28/28)	5 weeks	IC; CF
Purohit and Pradhan (2017)	India	Adolescence	Yoga program (*n* = 40/40)	Wait-list control / routine activities (*n* = 40/32)	12 weeks	CF
Jimenez Roldán and Chavarrias Olmedo (2025)	Spain	Childhood	Low-velocity resistance training (LVRT) (*n* = 13/13) High-velocity resistance training (HVRT) (*n* = 13/13)	Control group (CG) (*n* = 13/13)	8 weeks	IC; WM; CF
Solianik et al. (2021)	Lithuania	Later life	8-form Yang-style Tai Chi (*n* = 15/15)	No-intervention control / daily routine (*n* = 15/15)	10 weeks	IC; WM; CF
Stenman et al. (2017)	Finland	Adolescence	High-intensity interval training (HIT) (*n* = 12/9)	Usual-habits control (*n* = 13/10)	2 weeks	WM
Wang (2026)	China	Adolescence	High-intensity interval exercise (HIIE) group (*n* = 28/25) Moderate-intensity continuous exercise (MICE) group (*n* = 28/25)	Control group (*n* = 28/25)	12 weeks	IC; WM; CF
Wen et al. (2018)	China	Childhood	Mini-trampoline physical activity (*n* = 29/29)	Same preschool classes/care service (*n* = 28/28)	10 weeks	IC; WM; CF
Williams et al. (2022)	United Kingdom	Childhood	School-based sprint training intervention (*n* = 10/8)	Control group / regular daily routines (*n* = 10/8)	2 weeks	IC; WM
Yang et al. (2020)	China	Later life	Tai Chi Chuan group (*n* = 15/13)	General daily-activity control group (*n* = 15/13)	8 weeks	IC
Yu et al. (2025)	Taiwan	Later life	Exergame group (*n* = 49/46)	Control group (*n* = 51/45)	10 weeks	WM
Zhang et al. (2024)	China	Childhood	Moderate-intensity recreational gymnastics (*n* = 33/31)	Control group (*n* = 33/32)	12 weeks	IC; WM
Zhang et al. (2026)	China	Later life	Adapted tango dance group (*n* = 25/25)	Control group (*n* = 25/25)	12 weeks	IC; WM; CF
Zhao C. L. et al. (2022)	China	Later life	Active video game intervention group (IG) (*n* = 24/22)	Health education / control group (CG) (*n* = 24/16)	12 weeks	WM
Zhao C. X. et al. (2022)	China	Later life	Exergame training group (ET) (*n* = 25/21) Aerobic dancing training group (ADT) (*n* = 22/18)	No-training control group (CON) (*n* = 22/16)	12 weeks	IC; WM
Zhong et al. (2024)	China	Childhood	Volleyball physical training group (*n* = 40/38)	Daily routine control group (*n* = 40/38)	12 weeks	IC
Zinelabidine et al. (2022)	Tunisia	Childhood	Aerobic dance experimental group (EG) (*n* = 19/19)	No-PE control group (CG) (*n* = 22/22)	8 weeks	IC; CF

*n*, sample size; EF, executive function; IC, inhibitory control; WM, working memory; CF, cognitive flexibility. Arm-specific sample sizes are the actual numbers randomized/analyzed according to the source reports and are shown before any cluster-design effective-sample-size adjustment. Design-adjusted effective sample sizes used for effect-size variance or sampling-covariance calculations are not shown. Shared intervention or comparator arms in multi-arm trials are listed once within each study. Two trials contributed more than one independently extractable participant sample; the underlying arm-level randomized/analyzed counts are reported once here, while the independent-sample structure was retained in the analysis.

### 3.3 Risk of bias assessment

The risk-of-bias assessment included all 47 randomized controlled trials. Overall, 44 trials (93.6%) were rated as having some concerns, three (6.4%) as high risk, and none as low risk.

Across domains, low-risk judgments were recorded for 8 trials (17.0%) for the randomization process (D1), 7 (14.9%) for deviations from intended interventions (D2), 24 (51.1%) for missing outcome data (D3), 33 (70.2%) for outcome measurement (D4), and 3 (6.4%) for selection of the reported result (D5). The remaining judgments were predominantly some concerns; only D3 included high-risk ratings, affecting three trials (6.4%). Concerns were most frequent for selection of the reported result, followed by deviations from intended interventions and the randomization process, generally reflecting incomplete reporting rather than confirmed bias.

Hola et al. (2024), Zhao C. L. et al. (2022), and Lopes Filho et al. (2019) were rated at overall high risk because of high-risk D3 judgments. Trial-level judgments are shown in Figure 2, domain-level and overall distributions in Figure 3, and the sensitivity analysis excluding these trials in Section 3.8.

**Figure 2:**
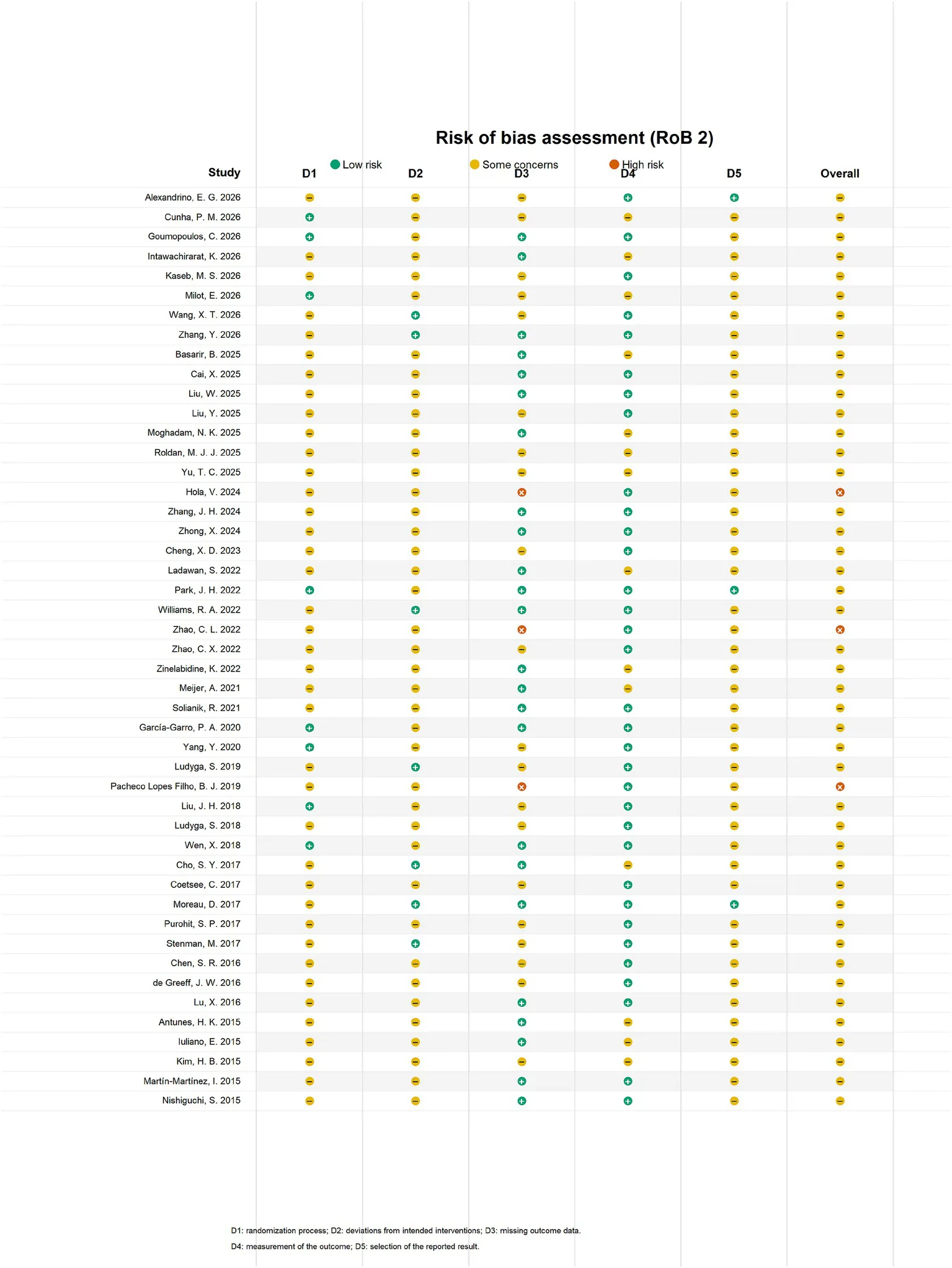
Study-level risk-of-bias judgments for the 47 included randomized controlled trials using the revised Cochrane Risk of Bias tool (RoB 2). D1, bias arising from the randomization process; D2, bias due to deviations from intended interventions; D3, bias due to missing outcome data; D4, bias in measurement of the outcome; D5, bias in selection of the reported result.

**Figure 3:**
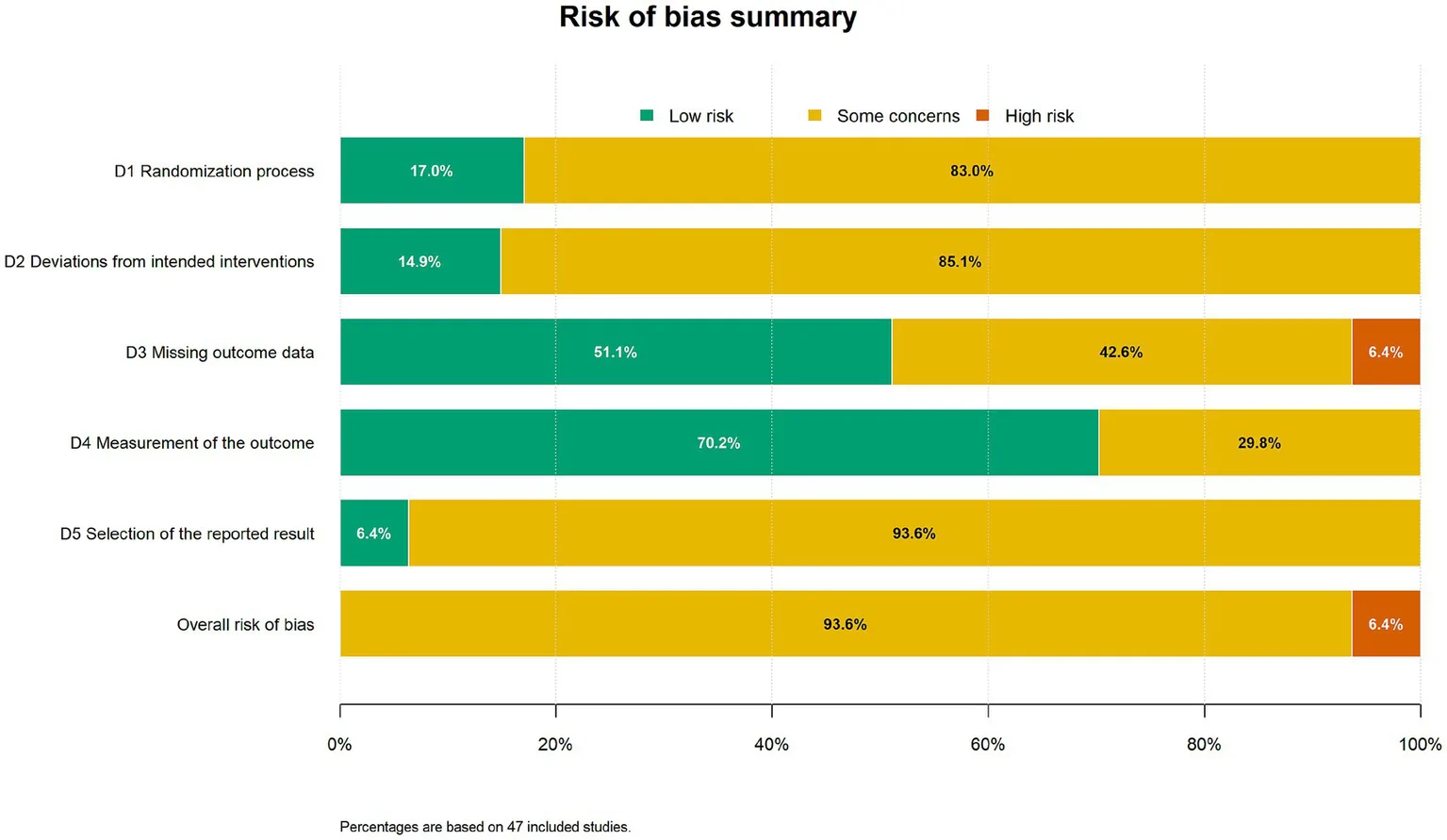
Distribution of domain-level and overall risk-of-bias judgments across the 47 included randomized controlled trials using the revised Cochrane Risk of Bias tool (RoB 2). D1, bias arising from the randomization process; D2, bias due to deviations from intended interventions; D3, bias due to missing outcome data; D4, bias in measurement of the outcome; D5, bias in selection of the reported result.

### 3.4 Overall effect and heterogeneity

The primary three-level random-effects model included 202 effect-size estimates from 47 randomized controlled trials, representing 49 independent participant samples and 4,195 actual participants. Structured exercise was associated with a positive average effect on executive function (Hedges’ *g* = 0.412, CR2 robust SE = 0.063), *t*(43.45) = 6.50, *p* < 0.001, with a Study_ID-clustered CR2 robust 95% CI of [0.284, 0.540].

Substantial heterogeneity remained at both modeled levels. The study-level variance component was 0.1324 and the residual effect-size-level component 0.0580. With a typical sampling variance of 0.0653, total multilevel *I*^2^ was 74.45%, comprising 51.79% at the study level and 22.66% at the effect-size level, indicating residual variation both between and within trials.

The 95% prediction interval ranged from −0.476 to 1.299. Thus, although the pooled mean was positive and its robust confidence interval excluded zero, true effects in future comparable intervention–comparator contrasts could range from negative or negligible to comparatively large positive effects. The pooled estimate should therefore be interpreted as an average across heterogeneous studies and outcomes rather than an effect expected uniformly across populations, interventions, outcomes, or settings (see Figure 4).

**Figure 4:**
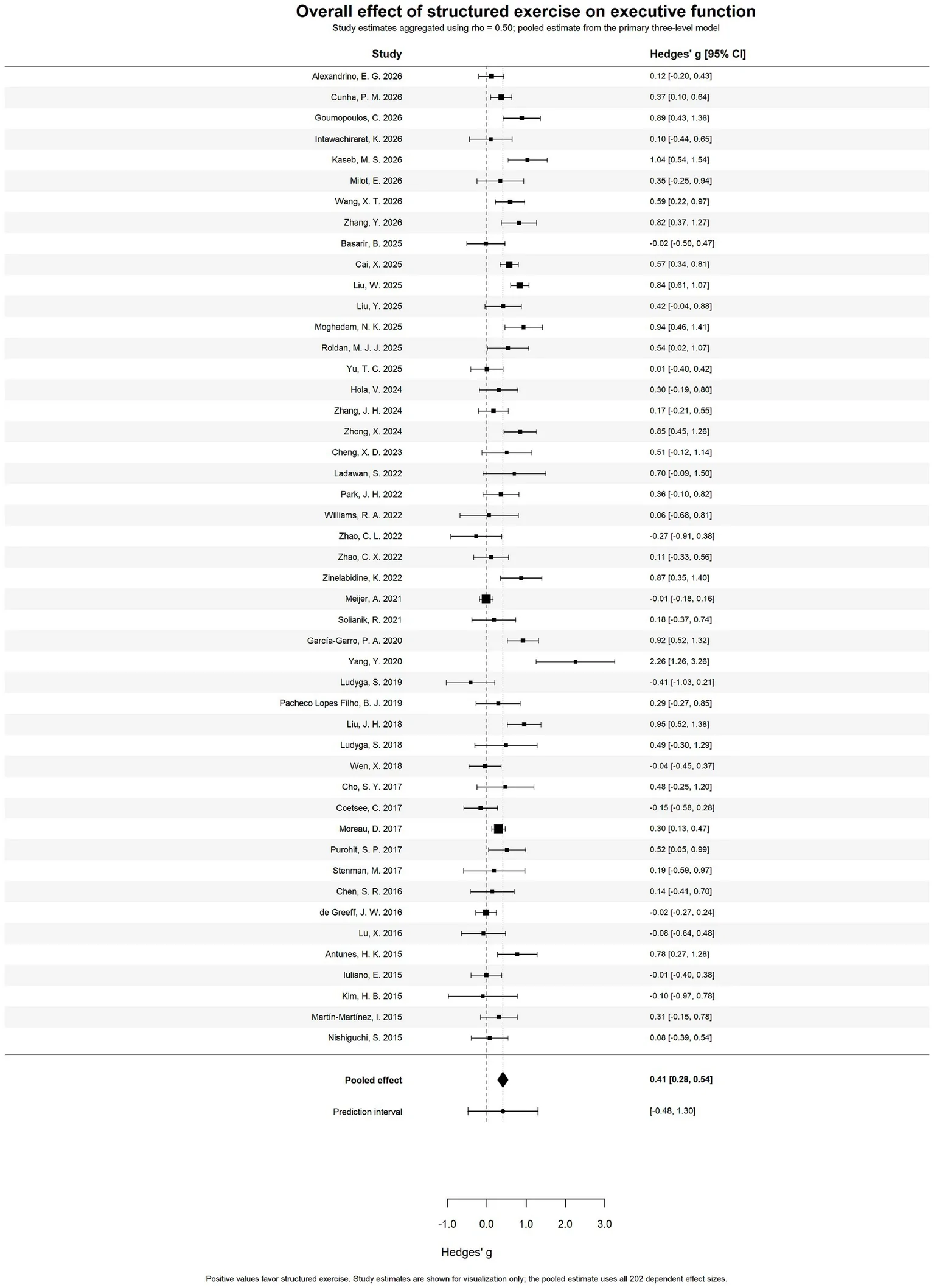
Forest plot of the overall effect of structured exercise on executive function. Individual study rows show generalized least-squares study-level estimates obtained by aggregating eligible dependent effect sizes within each trial under an assumed within-study correlation of ρ=0.50 ; these estimates are displayed for visualization only. The overall diamond represents the pooled mean and robust 95% confidence interval from the primary three-level random-effects model retaining all 202 effect-size estimates. Positive Hedges’ g values favor structured exercise over the comparator condition. CI, confidence interval.

### 3.5 Executive-function domain and developmental stage

#### 3.5.1 Executive-function domain

Inhibitory control comprised 89 effect-size estimates from 31 trials and 32 independent samples involving 3,183 participants; working memory, 51 estimates from 22 trials and 24 samples involving 2,703 participants; and cognitive flexibility, 62 estimates from 27 trials and 28 samples involving 2,035 participants. Because trials and samples could contribute to multiple domains, these participant counts overlapped and should not be summed.

Category-specific pooled effects were positive for all three domains, with Study_ID-clustered CR2 robust 95% confidence intervals excluding zero: inhibitory control, Hedges’ *g* = 0.361 (95% CI [0.174, 0.548]); working memory, *g* = 0.351 (95% CI [0.172, 0.530]); and cognitive flexibility, *g* = 0.521 (95% CI [0.394, 0.647]).

Although cognitive flexibility had the largest point estimate, the robust omnibus test did not indicate a statistically significant difference among domains, *F*(2, 16.82) = 3.34, *p* = 0.060. These numerical differences were therefore interpreted descriptively rather than as evidence of differential effects across executive-function domains.

#### 3.5.2 Developmental stage

The developmental-stage analysis included 90 effect-size estimates from 18 independent samples across 17 trials of children (*N* = 2,250), 30 estimates from 10 samples across nine adolescent trials (*N* = 756), and 82 estimates from 21 samples across 21 trials of older adults (*N* = 1,189). Because developmental stage was assigned at the independent-sample level, these participant counts were mutually exclusive and summed to 4,195. The adolescent evidence base was smaller than those for children and older adults.

Category-specific pooled effects were positive at all three stages, with Study_ID-clustered CR2 robust 95% confidence intervals excluding zero: children, Hedges’ *g* = 0.363 (95% CI [0.123, 0.603]); adolescents, *g* = 0.455 (95% CI [0.228, 0.682]); and older adults, *g* = 0.437 (95% CI [0.222, 0.652]).

The robust omnibus test did not indicate a statistically significant difference among stages, *F*(2, 21.92) = 0.19, *p* = 0.825. This finding should not be interpreted as evidence of equivalence, particularly given the smaller adolescent evidence base. Stage-specific covariance and leave-one-trial-out analyses are reported in Section 3.8, with estimates shown in Figure 5.

**Figure 5:**
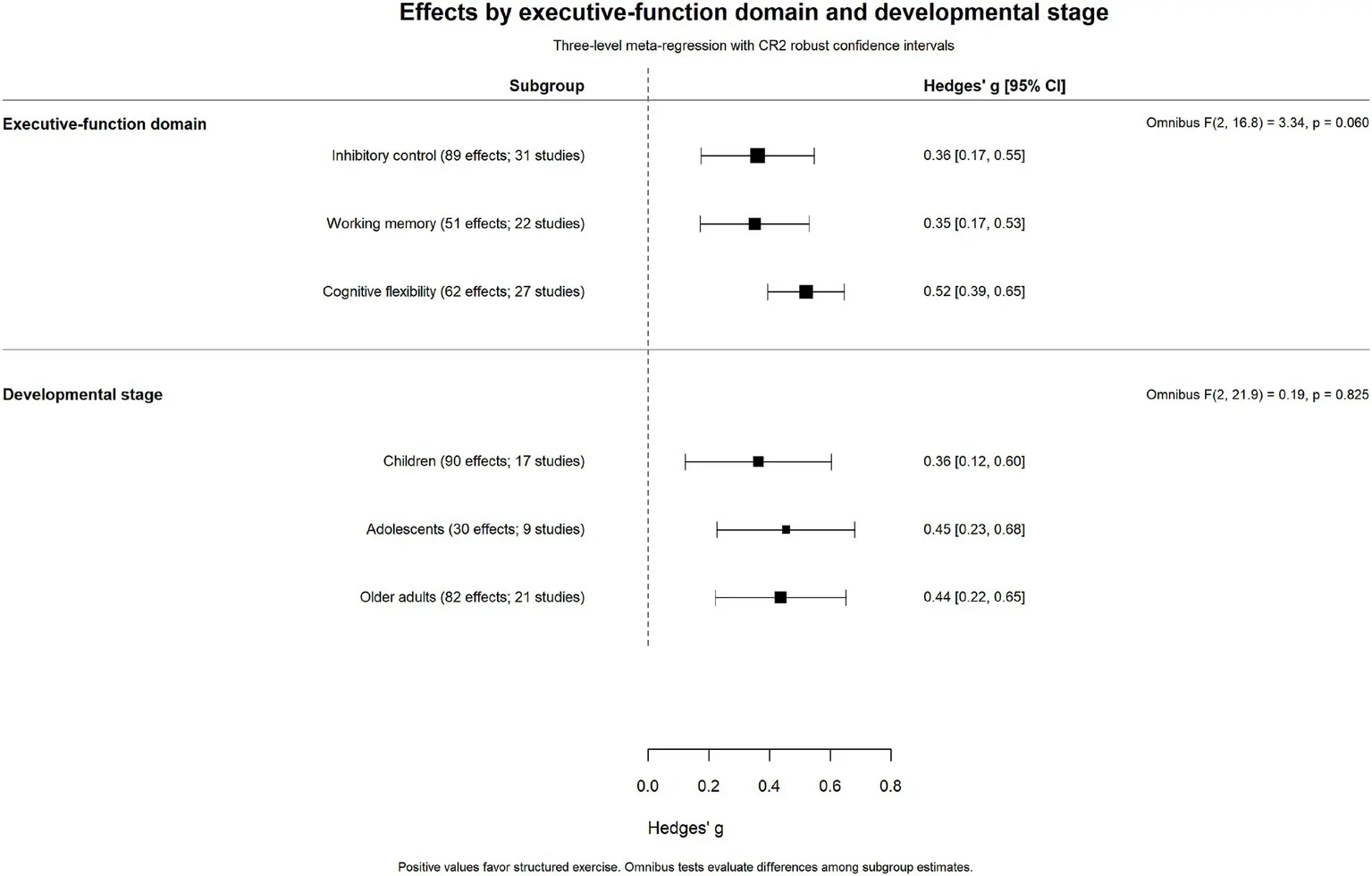
Pooled effects of structured exercise on executive function according to executive-function domain and developmental stage. **(A)** Estimates for inhibitory control, working memory, and cognitive flexibility. **(B)** Estimates for children, adolescents, and older adults. Points represent category-specific pooled Hedges’ g estimates obtained from no-intercept three-level meta-regression models, and horizontal lines represent study-clustered CR2 robust 95% confidence intervals. Positive values favor structured exercise over the comparator condition. Omnibus tests did not indicate statistically significant differences among executive-function domains, F(2,16.82)=3.34 , p=0.060 , or developmental stages, F(2,21.92)=0.19 , p=0.825 . CI, confidence interval.

### 3.6 Exercise skill characteristics

#### 3.6.1 Environmental predictability and cognitive engagement

For environmental predictability, stable environments contributed 130 effect-size estimates from 29 trials, semi-dynamic environments 48 estimates from 11 trials, and dynamic environments 24 estimates from nine trials; trial counts were not mutually exclusive. Category-specific pooled effects were positive: stable, Hedges’ *g* = 0.356 (95% CI [0.203, 0.509]); semi-dynamic, *g* = 0.581 (95% CI [0.305, 0.857]); and dynamic, *g* = 0.351 (95% CI [0.135, 0.566]). Although the semi-dynamic category had the largest point estimate, the Study_ID-clustered CR2 omnibus test was not statistically significant (*p* = 0.375); numerical differences were therefore interpreted descriptively.

For cognitive engagement, low-to-moderate and high-engagement interventions each contributed 101 effect-size estimates, from 20 and 29 trials, respectively. Pooled effects were positive for low-to-moderate engagement, *g* = 0.363 (95% CI [0.172, 0.553]), and high engagement, *g* = 0.447 (95% CI [0.279, 0.615]). The CR2 high-versus-low-to-moderate contrast was not statistically significant (*p* = 0.474).

Thus, neither environmental predictability nor cognitive engagement significantly moderated executive-function effects. Estimates are shown in Figure 6; adjusted analyses and covariance-sensitivity results are reported in Sections 3.7.3 and 3.8, respectively.

**Figure 6:**
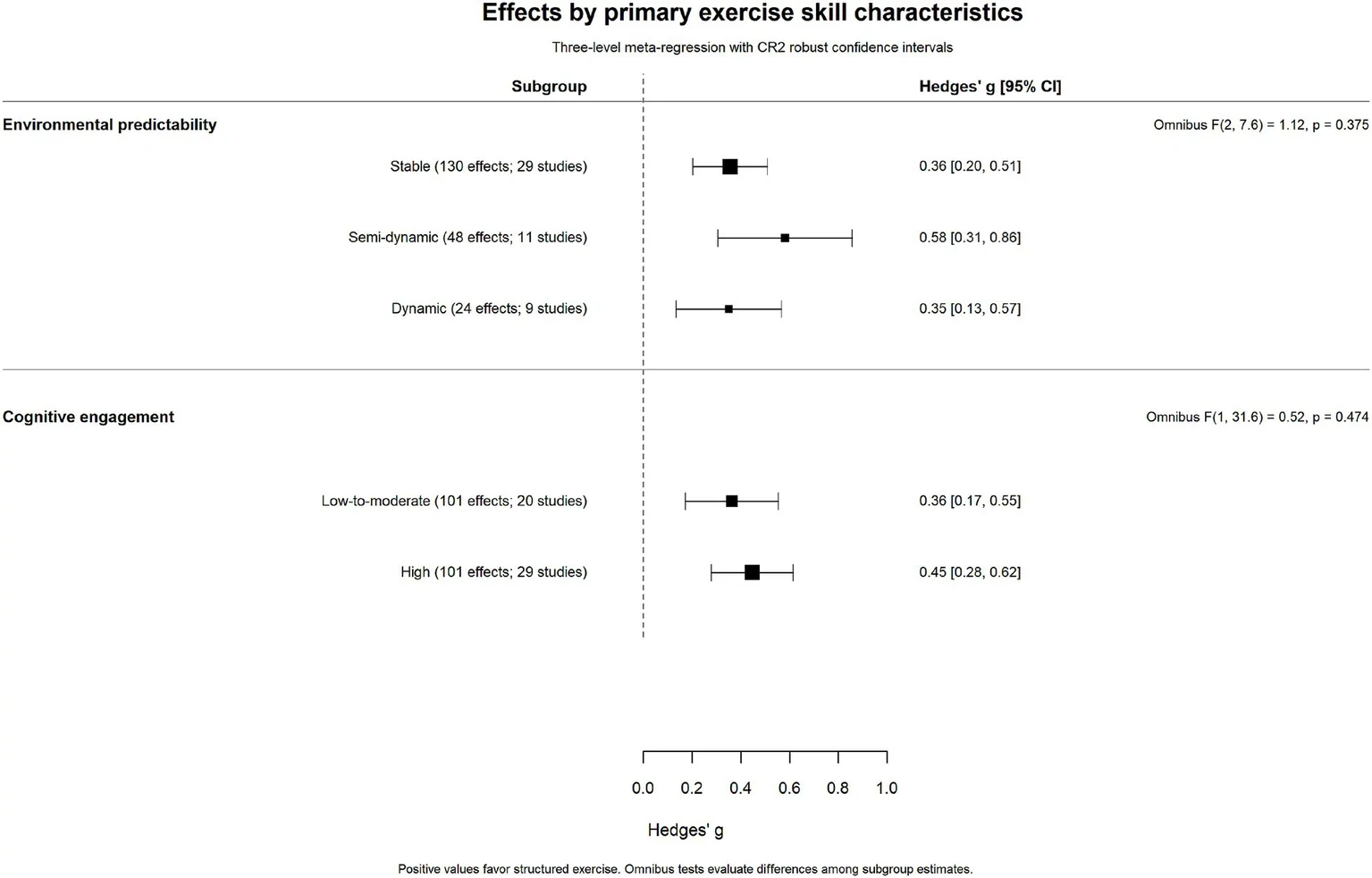
Pooled effects of structured exercise on executive function according to the principal and secondary skill-characteristic moderators in the final analytical framework. **(A)** Environmental predictability, classified as stable, semi-dynamic, or dynamic. **(B)** Cognitive engagement, classified as high or low-to-moderate. Points represent category-specific pooled Hedges’ g estimates obtained from no-intercept three-level meta-regression models, and horizontal lines represent study-clustered CR2 robust 95% confidence intervals. Positive values favor structured exercise over the comparator condition. The robust omnibus test for environmental predictability ( p=0.375 ) and the robust high-versus-low-to-moderate contrast for cognitive engagement ( p=0.474 ) did not indicate statistically significant between-category differences. CI, confidence interval.

#### 3.6.2 Coordination complexity and motor skill learning demand

Coordination complexity was examined as an exploratory moderator. Low-to-moderate-complexity interventions contributed 103 effect-size estimates from 22 trials and high-complexity interventions 99 estimates from 27 trials. Pooled effects were positive in both categories: low-to-moderate complexity, Hedges’ *g* = 0.398 (95% CI [0.223, 0.574]); and high complexity, *g* = 0.423 (95% CI [0.276, 0.570]). The Study_ID-clustered CR2 high-versus-low-to-moderate contrast was not statistically significant, Δ*g* = 0.024 (95% CI [−0.169, 0.218]), *p* = 0.779.

Motor skill learning demand was examined as a supplementary exploratory moderator. Low-to-moderate-demand interventions contributed 118 estimates from 26 trials and high-demand interventions 84 estimates from 23 trials. Pooled effects were *g* = 0.390 (95% CI [0.240, 0.540]) and *g* = 0.437 (95% CI [0.266, 0.608]), respectively; the contrast was not significant, Δ*g* = 0.047 (95% CI [−0.148, 0.243]), *p* = 0.591.

Neither characteristic significantly moderated effects, and non-significant contrasts should not be interpreted as equivalence. Motor skill learning demand was considered supplementary because of its conceptual and empirical overlap with coordination complexity. Estimates are shown in Figure 7; covariance-sensitivity results are reported in Section 3.8.

**Figure 7:**
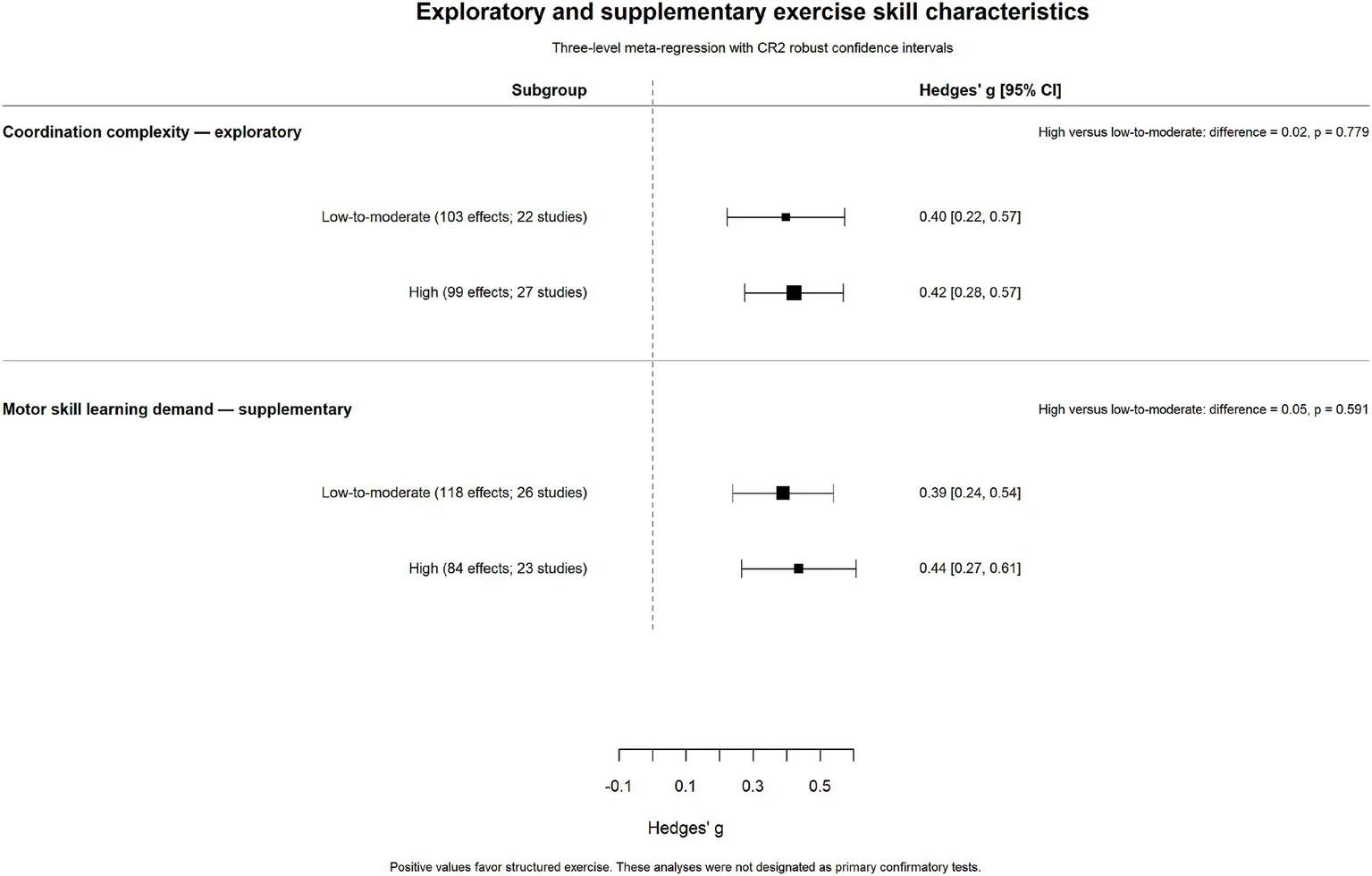
Pooled effects of structured exercise on executive function according to the exploratory and supplementary skill-characteristic moderators. **(A)** Coordination complexity, classified as high or low-to-moderate. **(B)** Motor skill learning demand, classified as high or low-to-moderate. Points represent category-specific pooled Hedges’ g estimates obtained from no-intercept three-level meta-regression models, and horizontal lines represent study-clustered CR2 robust 95% confidence intervals. Positive values favor structured exercise over the comparator condition. The direct high-versus-low-to-moderate contrasts did not indicate statistically significant differences for coordination complexity ( Δg=0.024 , 95% CI [−0.169, 0.218], p=0.779 ) or motor skill learning demand ( Δg=0.047 , 95% CI [−0.148, 0.243], p=0.591 ). CI, confidence interval.

### 3.7 Additional moderator and adjusted analyses

#### 3.7.1 Intervention duration

Intervention duration was available for all 61 eligible structured-exercise arms (2–44 weeks), and all 202 effect-size estimates from 47 trials were retained in the full-range analyses.

In the linear model, duration was not significantly associated with effect magnitude under either the primary diagonal structure (Δ*g* = −0.034 per additional 4 weeks, 95% CI [−0.168, 0.099], *p* = 0.401) or the non-diagonal structure with ρ = 0.50 (Δ*g* = −0.037, 95% CI [−0.166, 0.093], *p* = 0.344). Satterthwaite-adjusted denominator degrees of freedom were 2.14 and 1.98, respectively, indicating limited effective information. Results were similarly non-significant for a doubling of duration: Δ*g* = −0.031 (95% CI [−0.245, 0.184], *p* = 0.734) under the diagonal structure and −0.046 (95% CI [−0.258, 0.165], *p* = 0.611) at ρ = 0.50.

Because only two trials exceeded 16 weeks, a *post hoc* analysis restricted duration to 2–16 weeks, retaining 195 estimates from 45 trials. The estimated change per additional 4 weeks remained non-significant under the diagonal structure (Δ*g* = −0.059, 95% CI [−0.234, 0.116], *p* = 0.481) and at ρ = 0.50 (Δ*g* = −0.064, 95% CI [−0.235, 0.107], *p* = 0.435); duration-doubling estimates were likewise non-significant.

Overall, neither specification provided reliable evidence of duration-related moderation. However, the uneven duration distribution, only two trials beyond 16 weeks, very small effective degrees of freedom, and wide confidence intervals limit precision; these findings do not establish that duration is unimportant or that shorter and longer interventions are equivalent. Complete results are reported in Supplementary Table S3.

#### 3.7.2 Comparator type

Comparator type was examined as an exploratory moderator. Passive/usual-activity/usual-care comparators contributed 116 effect-size estimates from 29 trials, active nonexercise attention/low-demand comparators 30 estimates from nine trials, and usual physical education/routine physical activity comparators 56 estimates from 10 trials; trial counts were not mutually exclusive.

Category-specific pooled effects were Hedges’ *g* = 0.448 (95% CI [0.274, 0.621]) for passive/usual-activity/usual-care comparators, *g* = 0.229 (95% CI [−0.033, 0.490]) for active nonexercise attention/low-demand comparators, and *g* = 0.451 (95% CI [0.168, 0.734]) for usual physical education/routine physical activity comparators.

Although the active nonexercise category had the smallest point estimate, the robust omnibus test was not statistically significant under either the primary diagonal structure (*p* = 0.262) or the non-diagonal structure with ρ = 0.50 (*p* = 0.270). Comparator type was not randomly assigned across trials; these aggregate-level comparisons therefore do not establish that passive controls inflate effects or active controls attenuate them. The passive/usual-activity/usual-care sensitivity analysis is reported in Section 3.8.

#### 3.7.3 Adjusted skill-characteristic models

Environmental predictability and cognitive engagement were re-estimated in separate covariate-adjusted three-level meta-regression models including executive-function domain, developmental stage, and comparator type. Both retained all 202 effect-size estimates from 47 trials and were conducted as robustness analyses rather than causal adjustment models.

For environmental predictability, the adjusted robust omnibus test was not statistically significant under either the primary diagonal structure, *F*(2, 7.02) = 1.16, *p* = 0.366, or the non-diagonal structure with ρ = 0.50, *F*(2, 10.19) = 1.59, *p* = 0.250. The small denominator degrees of freedom indicated limited effective information after adjustment.

For cognitive engagement, the adjusted high-versus-low-to-moderate contrast was Δ*g* = 0.095 (CR2 robust SE = 0.118), *t*(27.97) = 0.81, 95% CI [−0.146, 0.337], *p* = 0.427 under the diagonal structure, and Δg = 0.110 (CR2 robust SE = 0.116), *t*(29.08) = 0.95, 95% CI [−0.126, 0.347], *p* = 0.348 at ρ = 0.50.

These adjusted analyses were consistent with the unadjusted results: neither characteristic showed reliable moderation. Residual confounding remains possible, so the estimates should not be interpreted causally. Coordination complexity and motor skill learning demand were not included because of overlap, limited study-level information, and overparameterization risk (see Table 2).

**Table 2:** Summary of the primary, subgroup, moderator, and adjusted meta-analytic results.

Analysis	Category or Contrast	Effect sizes, n	Trials, n	Participants, n	Estimate	Robust 95% CI	Robust test
Overall effect	All outcomes	202	47	4,195	*g* = 0.412	[0.284, 0.540]	*t*(43.45) = 6.50, *p* < 0.001
Executive-function domain	Inhibitory control	89	31	3,183	*g* = 0.361	[0.174, 0.548]	
Executive-function domain	Working memory	51	22	2,703	*g* = 0.351	[0.172, 0.530]	
Executive-function domain	Cognitive flexibility	62	27	2,035	*g* = 0.521	[0.394, 0.647]	
Executive-function domain	Omnibus moderator test	202	47	4,195	—	—	*F*(2, 16.82) = 3.34, *p* = 0.060
Developmental stage	Children	90	17	2,250	*g* = 0.363	[0.123, 0.603]	
Developmental stage	Adolescents	30	9	756	*g* = 0.455	[0.228, 0.682]	
Developmental stage	Older adults	82	21	1,189	*g* = 0.437	[0.222, 0.652]	
Developmental stage	Omnibus moderator test	202	47	4,195	—	—	*F*(2, 21.92) = 0.19, *p* = 0.825
Environmental predictability	Stable	130	29	—	*g* = 0.356	[0.203, 0.509]	
Environmental predictability	Semi-dynamic	48	11	—	*g* = 0.581	[0.305, 0.857]	
Environmental predictability	Dynamic	24	9	—	*g* = 0.351	[0.135, 0.566]	
Environmental predictability	Omnibus moderator test	202	47	—	—	—	*p* = 0.375
Cognitive engagement	Low-to-moderate	101	20	—	*g* = 0.363	[0.172, 0.553]	
Cognitive engagement	High	101	29	—	*g* = 0.447	[0.279, 0.615]	
Cognitive engagement	High vs. low-to-moderate	202	47	—	—	—	*p* = 0.474
Coordination complexity	Low-to-moderate	103	22	—	*g* = 0.398	[0.223, 0.574]	
Coordination complexity	High	99	27	—	*g* = 0.423	[0.276, 0.570]	
Coordination complexity	High vs. low-to-moderate	202	47	—	Δ*g* = 0.024	[−0.169, 0.218]	*p* = 0.779
Motor skill learning demand	Low-to-moderate	118	26	—	*g* = 0.390	[0.240, 0.540]	
Motor skill learning demand	High	84	23	—	*g* = 0.437	[0.266, 0.608]	
Motor skill learning demand	High vs. low-to-moderate	202	47	—	Δ*g* = 0.047	[−0.148, 0.243]	*p* = 0.591
Comparator type	Passive/usual activity/usual care	116	29	—	*g* = 0.448	[0.274, 0.621]	
Comparator type	Active nonexercise attention/low-demand	30	9	—	*g* = 0.229	[−0.033, 0.490]	
Comparator type	Usual PE or routine physical activity	56	10	—	*g* = 0.451	[0.168, 0.734]	
Comparator type	Omnibus moderator test	202	47	—	—	—	*p* = 0.262
Adjusted skill model	Environmental predictability omnibus	202	47	—	—	—	*F*(2, 7.02) = 1.16, *p* = 0.366
Adjusted skill model	Cognitive engagement: high vs. low-to-moderate	202	47	—	Δ*g* = 0.095	[−0.146, 0.337]	*t*(27.97) = 0.81, *p* = 0.427

Category-specific estimates are pooled Hedges’ *g*-values; Δ*g* denotes direct between-category differences. Robust inference used Study_ID-clustered CR2 variance estimation. Participant counts are actual analyzed participants. Domain-specific participant counts overlap and should not be summed, whereas developmental-stage participant counts are mutually exclusive and sum to 4,195. Participant counts are omitted for intervention-arm- and comparator-level moderator rows because shared participants may contribute to multiple contrasts or categories within trials. Trial counts across categories may also overlap. The primary overall model had total *I*^2^ = 74.45% and a 95% prediction interval of [−0.476, 1.299]. Complete intervention-duration and sensitivity analyses are reported in Supplementary Tables S3, S4, respectively. CI, confidence interval; PE, physical education.

### 3.8 Sensitivity and robustness analyses

The positive pooled mean was generally robust across sensitivity and robustness analyses. Replacing the primary diagonal sampling-variance matrix with non-diagonal matrices assuming within-sample correlations of ρ = 0.30, 0.50, and 0.70 yielded Hedges’ *g* = 0.405 (CR2 robust 95% CI [0.278, 0.531]), 0.400 ([0.273, 0.526]), and 0.395 ([0.267, 0.522]), respectively, compared with *g* = 0.412 ([0.284, 0.540]) under the diagonal specification. The largest relative change was approximately 4.2%, and all robust confidence intervals excluded zero.

Heterogeneity measures were more sensitive to covariance assumptions. Total multilevel *I*^2^ increased from 74.45% under the diagonal specification to 77.66%, 82.31%, and 88.46% at ρ = 0.30, 0.50, and 0.70, respectively, while corresponding 95% prediction intervals remained wide and crossed zero. The increase in *I*^2^ primarily reflected changes in scenario-specific typical sampling variance rather than comparable increases in the absolute heterogeneity variance components; therefore, stronger assumed correlations should not be interpreted as evidence that underlying heterogeneity itself increased substantially.

Covariance sensitivity analyses for executive-function domain, developmental stage, environmental predictability, cognitive engagement, coordination complexity, and motor skill learning demand did not alter moderator conclusions. None became statistically significant across ρ = 0.30–0.70. For executive-function domain, the omnibus *p* value changed from 0.060 under the diagonal specification to 0.077, 0.092, and 0.107, respectively.

Stage-specific intercept-only models also showed limited sensitivity to covariance assumptions. Under the diagonal structure, pooled estimates were *g* = 0.368 for children, 0.460 for adolescents, and 0.443 for older adults; maximum relative changes across alternative covariance assumptions were approximately 7.52%, 0.52%, and 1.57%, respectively. Adolescent models had Satterthwaite-adjusted degrees of freedom of approximately 6.25–7.00. Robust confidence intervals excluded zero across these models, but prediction intervals crossed zero in all three stages, indicating stability of average estimates rather than uniform positive effects across future comparable settings.

Leave-one-trial-out analyses further supported overall stability. Across 47 overall models, pooled effects ranged from *g* = 0.390 to 0.426, with all CR2 robust 95% confidence intervals excluding zero. Removing Study ID NO.32 produced the largest change, reducing the estimate to *g* = 0.390, approximately 5.35% below the primary pooled estimate.

Stage-specific leave-one-trial-out analyses showed greater variation in children and adolescents than in older adults. Maximum relative changes were 18.46% for children, 20.79% for adolescents, and 9.98% for older adults. Adolescent estimates ranged from *g* = 0.398 to 0.555, and the minimum Satterthwaite-adjusted degrees of freedom was 4.73. All stage-specific estimates remained positive with robust confidence intervals excluding zero, although effect magnitudes, particularly in adolescents, were less stable.

Additional sensitivity analyses produced similar overall estimates. Alternative ICC assumptions yielded *g* ≈ 0.412, and Morris change-score analyses yielded *g* ≈ 0.425–0.426. Baseline-imbalance exclusions produced estimates of *g* = 0.415 and 0.446 at absolute standardized-mean-difference thresholds of 0.80 and 0.50, respectively. Excluding the three high-risk trials yielded *g* = 0.427; restricting to high-confidence coded arms yielded *g* = 0.426, with environmental predictability and cognitive engagement remaining non-significant. Restriction to passive/usual-activity/usual-care comparators yielded *g* = 0.457, and exclusion of the trial requiring an assumed ICC yielded *g* = 0.411.

Across the 15 pooled-effect sensitivity scenarios shown in Figure 8, estimates ranged from *g* = 0.395 to 0.457, and all CR2 robust 95% confidence intervals excluded zero. Overall, the positive average effect and non-significant moderator findings were generally stable to the analytical assumptions and study restrictions examined. However, wide prediction intervals, greater stage-specific leave-one-trial-out variation in children and particularly adolescents, and limited effective degrees of freedom indicate that robustness of the average direction should not be interpreted as uniformity or precise stability of effect magnitude. Complete results are reported in Supplementary Table S4.

**Figure 8:**
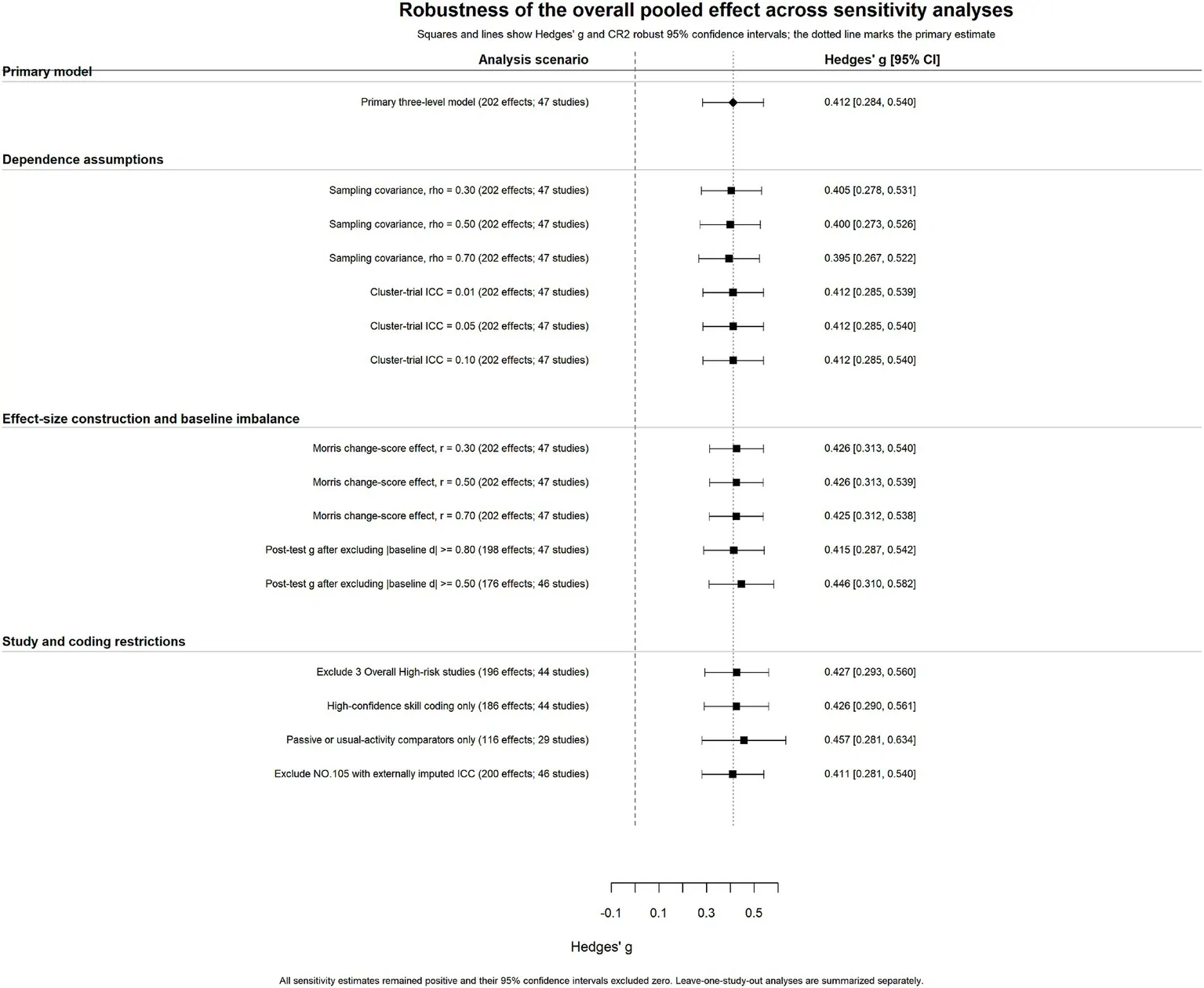
Sensitivity and robustness analyses of the overall pooled effect of structured exercise on executive function. Point estimates represent pooled Hedges’ g values, and horizontal lines represent study-clustered CR2 robust 95% confidence intervals. The figure presents the primary three-level model and 15 pooled-effect sensitivity scenarios examining alternative sampling-covariance assumptions, cluster-randomized-trial ICC values, effect-size construction, baseline-imbalance exclusions, risk-of-bias restrictions, skill-coding confidence, comparator category, and exclusion of the trial requiring an assumed ICC. Positive values favor structured exercise over the comparator condition. CR2, small-sample-corrected cluster-robust variance estimation; ICC, intracluster correlation coefficient.

### 3.9 Assessment of small-study effects and potential publication bias

The sample-size-based multilevel diagnostic did not show a statistically significant association between effect magnitude and study-size-related imprecision under either the primary diagonal structure (β = −0.152, 95% CI [−1.509, 1.204], *p* = 0.815) or the non-diagonal structure with ρ = 0.50 (β = −0.219, 95% CI [−1.643, 1.206], *p* = 0.749).

In contrast, the Egger-type multilevel model using the sampling standard error of Hedges’ *g* yielded significant positive coefficients under both the diagonal structure (β = 2.913, 95% CI [0.323, 5.503], *p* = 0.030) and ρ = 0.50 (β = 3.016, 95% CI [0.418, 5.614], *p* = 0.025). These findings were interpreted cautiously because the sampling standard error of a standardized mean difference partly depends on the effect estimate itself, creating potential mathematical coupling (Pustejovsky and Rodgers, 2019).

Study-level diagnostics based on 47 generalized least-squares aggregated trial estimates were not statistically significant: the Egger asymmetry intercept was 0.495 (95% CI [−0.982, 1.971], *p* = 0.503), and the Begg–Mazumdar test yielded Kendall’s τ = 0.032 (*p* = 0.757). An auxiliary REML model of these aggregated estimates yielded Hedges’ *g* = 0.376 (95% CI [0.255, 0.497]), *I*^2^ = 69.86%, and a 95% prediction interval of [−0.259, 1.011], but did not replace the primary three-level analysis.

Overall, the diagnostics were inconsistent. Because asymmetry may reflect heterogeneity, methodological differences, sampling variation, or mathematical coupling rather than publication bias alone (Sterne et al., 2011), the significant findings do not confirm publication bias, while the non-significant findings do not exclude it. Publication bias therefore remained uncertain. Figure 9 presents the contour-enhanced funnel plot.

**Figure 9:**
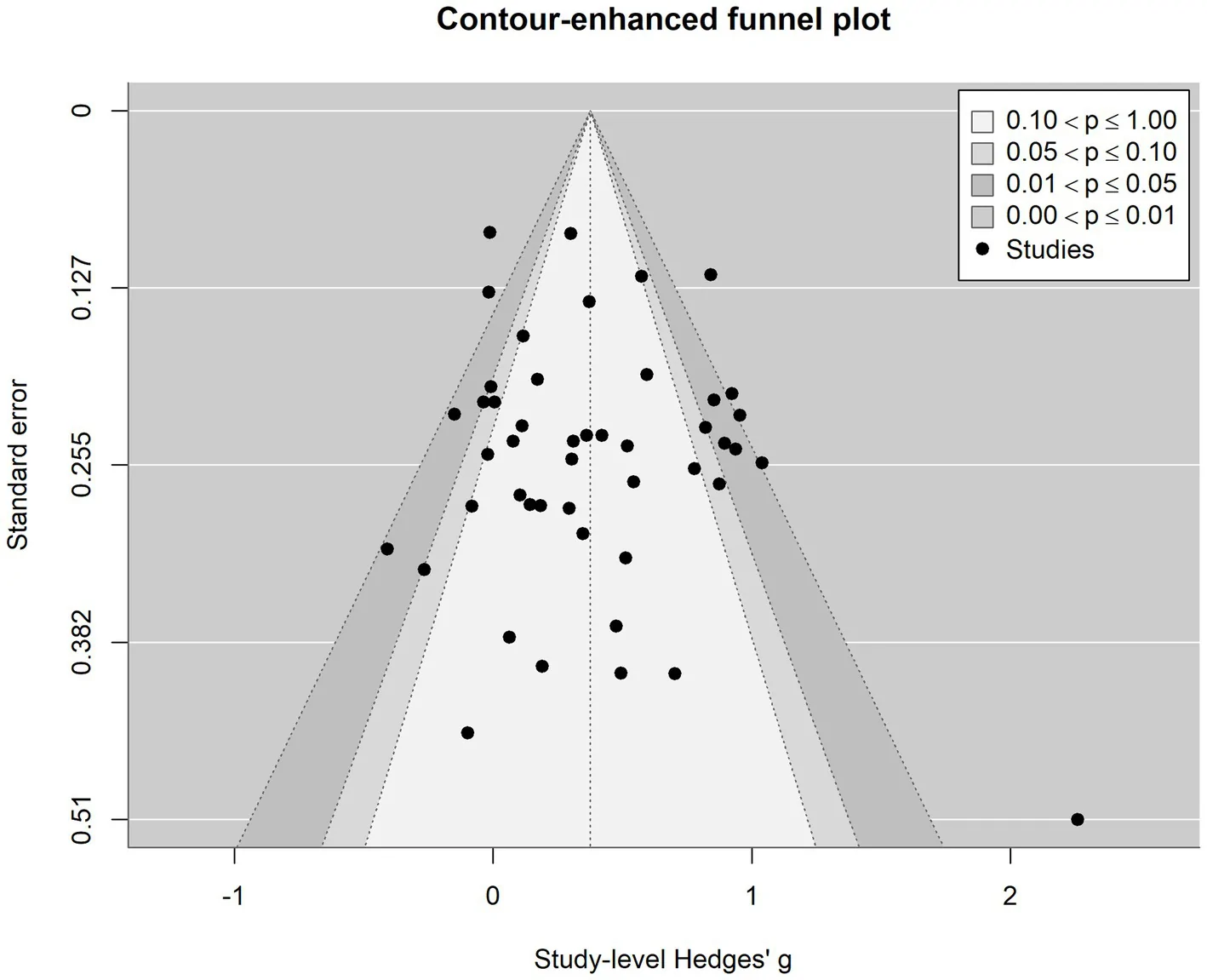
Contour-enhanced funnel plot of the 47 aggregated study-level estimates. Multiple eligible effect-size estimates within each trial were combined using generalized least-squares aggregation under an assumed within-study correlation of ρ = 0.50. Each point represents one aggregated study-level Hedges’ *g* estimate plotted against its standard error. Shaded contours indicate conventional two-sided statistical-significance regions. Positive effect sizes favor structured exercise over the comparator condition. The plot was interpreted descriptively together with the dependency-aware multilevel diagnostics and aggregated study-level asymmetry tests and was not treated as standalone evidence for or against publication bias.

### 3.10 Certainty of evidence

Certainty of evidence was rated as low for overall executive-function performance and separately for inhibitory control, working memory, and cognitive flexibility. Each body of randomized evidence was downgraded one level for serious risk of bias and one level for serious inconsistency. The risk-of-bias downgrade reflected the absence of trials at overall low risk, widespread concerns, and three trials at high risk, whereas the inconsistency downgrade reflected substantial unexplained heterogeneity and prediction intervals crossing the line of no effect.

No additional downgrade was applied for publication bias, but this should not be interpreted as evidence of its absence. Small-study-effect diagnostics were inconsistent: sample-size-based multilevel and aggregated study-level analyses were non-significant, whereas standard-error-based multilevel models yielded significant associations potentially influenced by mathematical coupling. Restriction to English-language, formally published journal articles and the absence of grey-literature searching further limited evidence completeness. Publication bias therefore remained uncertain, but the available evidence was considered insufficient to justify an additional GRADE downgrade. Detailed judgments are reported in Supplementary Table S6.

## 4 Discussion

### 4.1 Summary of main findings

This systematic review and three-level meta-analysis synthesized 202 effect-size estimates from 47 randomized controlled trials representing 49 independent participant samples and 4,195 actual participants across childhood, adolescence, and later life. Structured exercise was associated with a positive average effect on executive function (Hedges’ *g* = 0.412, Study_ID-clustered CR2 robust 95% CI [0.284, 0.540]), and this estimate changed only modestly across alternative sampling-covariance assumptions and other sensitivity analyses.

Substantial residual heterogeneity nevertheless remained. Total multilevel *I*^2^ was 74.45% under the primary model, and the 95% prediction interval ranged from −0.476 to 1.299 and continued to span zero under alternative covariance assumptions. Thus, the findings support a positive average effect but not a uniform benefit across populations, interventions, outcomes, or comparable future settings.

Positive pooled estimates were observed for all three executive-function domains and developmental stages, but neither domain nor developmental stage significantly moderated effects. Stage-specific estimates were generally stable across covariance assumptions, although leave-one-trial-out analyses showed greater variation in children and particularly adolescents, whose evidence base comprised only nine trials. Non-significant stage differences should therefore not be interpreted as equivalence.

Environmental predictability, cognitive engagement, coordination complexity, motor skill learning demand, intervention duration, and comparator type also showed no statistically significant moderation. These moderator conclusions were generally stable across the relevant covariance-sensitivity analyses; covariate-adjusted models of environmental predictability and cognitive engagement were also consistent with the unadjusted results. Sparse categories, uneven distributions, and limited effective degrees of freedom nevertheless constrain interpretation, and non-significant findings do not establish equivalence or irrelevance.

Risk of bias and inconsistency remained important limitations. No trial was rated at overall low risk, 44 had some concerns, and three were at high risk. Small-study-effect diagnostics were inconsistent and did not resolve the possibility of publication bias. Accordingly, certainty of evidence was rated as low for overall executive function and for each executive-function domain.

### 4.2 Interpretation of the overall effect and heterogeneity

The positive pooled effect is broadly consistent with previous reviews reporting beneficial associations of physical activity or structured exercise with cognition in children and adolescents (Donnelly et al., 2016; Alvarez-Bueno et al., 2017; De Greeff et al., 2018) and in middle-aged and older adults (Northey et al., 2018). The present review extends this literature by synthesizing evidence across childhood, adolescence, and later life within a dependency-aware framework that retained multiple eligible outcomes and intervention–comparator contrasts from the same trials.

The pooled Hedges’ *g* of 0.412 should be interpreted as a standardized average difference across heterogeneous executive-function measures rather than as a universal indicator of practical or clinical importance. Outcomes varied in task, metric, scoring convention, reliability, and scale, and no validated minimally important difference applied across them. Generic labels such as small, moderate, or large are therefore of limited value here. The estimate indicates better executive-function performance on average in structured-exercise groups, but not a uniform benefit across participants, tasks, or settings.

Several mechanisms could plausibly contribute to this average effect. Exercise may influence cerebral perfusion, cardiovascular and metabolic function, neurotrophic signaling, neural plasticity, and neural efficiency (Hillman et al., 2008; Erickson et al., 2011; Gomez-Pinilla and Hillman, 2013; Voss et al., 2013). Structured exercise may also engage effort regulation, performance monitoring, response inhibition, updating, and adaptation to changing demands (Best, 2010; Pesce, 2012; Tomporowski et al., 2015; Diamond and Ling, 2016). These remain plausible mechanisms rather than demonstrated mediators because mediation was not directly tested.

Substantial residual heterogeneity remained at both modeled levels. Under the primary diagonal specification, study-level and residual within-trial effect-size variance components were 0.1324 and 0.0580, corresponding to *I*^2^ values of 51.79 and 22.66%, with total multilevel *I*^2^ = 74.45%. Thus, variability arose both between trials and among outcomes or contrasts within trials. Differences in participants, intervention delivery, adherence, supervision, comparators, executive-function tasks, and scoring methods may have contributed, but variance decomposition does not identify causes, and aggregate-data moderator analyses cannot fully resolve them (Thompson and Higgins, 2002).

Covariance sensitivity analyses further qualify interpretation of heterogeneity. Total *I*^2^ increased from 74.45% under the diagonal specification to 77.66–88.46% as assumed within-sample correlations increased from ρ = 0.30 to 0.70, whereas the pooled effect changed only modestly from *g* = 0.412 to 0.395. This rise in *I*^2^ primarily reflected changes in scenario-specific sampling variance rather than comparable increases in the absolute heterogeneity variance components and should not be interpreted as increased underlying heterogeneity. The 95% prediction interval under the primary model was −0.476 to 1.299 and continued to span zero under alternative covariance assumptions. The robust confidence interval therefore supports a positive mean effect, whereas the prediction interval indicates substantial uncertainty in future comparable contrasts (Higgins et al., 2009; IntHout et al., 2016). Structured exercise may improve executive function on average, but a similar positive effect cannot be assumed across all populations, interventions, outcomes, or settings.

### 4.3 Executive-function domains and developmental stages

Positive pooled estimates were observed for inhibitory control, working memory, and cognitive flexibility, indicating that the average association between structured exercise and executive-function performance was not confined to one domain. Because trials and samples could contribute outcomes to multiple domains, participant populations overlapped. Although cognitive flexibility had the largest point estimate, the omnibus moderator test was not statistically significant; this difference should therefore be interpreted descriptively rather than as evidence of greater responsiveness.

The absence of significant domain moderation is consistent with models in which inhibitory control, working memory, and cognitive flexibility are partly separable but share common executive-control processes (Miyake et al., 2000; Diamond, 2013; Friedman and Miyake, 2017). Exercise may engage several of these processes simultaneously through inhibition, rule updating, switching, error monitoring, and adaptation. However, executive-function tasks are not process-pure (Miyake et al., 2000; Karr et al., 2018), and apparent domain differences may also reflect processing speed, visuomotor demands, task difficulty, reliability, scoring, or study composition. Covariance sensitivity analyses supported this interpretation: the omnibus *p* value changed from 0.060 under the diagonal specification to 0.077, 0.092, and 0.107 at ρ = 0.30, 0.50, and 0.70, respectively, without crossing the conventional significance threshold.

Positive pooled estimates were also observed for children, adolescents, and older adults, but developmental stage did not significantly moderate effects. This should not be interpreted as equivalence across age groups. The evidence base comprised 17 trials and 2,250 participants in children, nine trials and 756 participants in adolescents, and 21 trials and 1,189 participants in older adults. The smaller adolescent evidence base limited precision.

Stage-specific robustness analyses further qualified these findings. Across ρ = 0.30–0.70, maximum relative changes in pooled estimates were approximately 7.52% for children, 0.52% for adolescents, and 1.57% for older adults, indicating limited sensitivity to assumed within-sample correlation. Nevertheless, prediction intervals crossed zero in all three stages. Leave-one-trial-out analyses also preserved positive pooled estimates with robust confidence intervals excluding zero, but effect magnitudes varied more in children and adolescents. Maximum relative changes were 18.46%, 20.79%, and 9.98% for children, adolescents, and older adults, respectively; adolescent estimates ranged from approximately *g* = 0.398 to 0.555, with the minimum Satterthwaite-adjusted degrees of freedom equal to 4.73. Thus, the direction of the adolescent estimate was stable, but its magnitude remained sensitive to the composition of a small evidence base.

Developmental mechanisms may also differ. During childhood and adolescence, maturation of executive-control systems may interact with aerobic fitness, arousal, cognitively engaging movement, motor learning, and behavioral regulation (Hillman et al., 2008; Best, 2010; Best and Miller, 2010; Diamond, 2013; Tomporowski et al., 2015; Donnelly et al., 2016; De Greeff et al., 2018). In later life, cardiovascular and metabolic function, cerebral perfusion, neuroplasticity, and compensatory neural efficiency may be more salient in age-related change (Hillman et al., 2008; Park and Reuter-Lorenz, 2009; Salthouse, 2010; Erickson et al., 2011; Voss et al., 2013; Northey et al., 2018). These remain plausible mechanisms rather than demonstrated mediators.

More broadly, developmental stage represents an aggregate characteristic of different trial sets rather than a randomized comparison of age groups. The stages differed in intervention setting, exercise content, comparator type, duration, and executive-function measurement. Young and middle-aged adults were not eligible, and findings should not be generalized to them. The planned domain-by-developmental-stage interaction was not fitted because several cross-classified cells contained too few independent trials for stable study-clustered inference. More comparable, adequately powered age-specific trials are needed to test developmental differences directly.

### 4.4 Exercise skill characteristics

A distinctive feature of this review was the examination of qualitative exercise characteristics beyond conventional modality and duration labels. Environmental predictability, cognitive engagement, coordination complexity, and motor skill learning demand were examined because changing environments, decision-making, coordination, and skill acquisition may recruit executive-control processes (Best, 2010; Pesce, 2012; Tomporowski et al., 2015; Diamond and Ling, 2016). Coding these dimensions at the intervention-arm level characterized recurring intervention demands rather than assuming that modality labels represent fixed cognitive or motor challenges.

None of the four coded characteristics significantly moderated executive-function effects. Semi-dynamic environments and high cognitive engagement showed numerically larger pooled estimates than their comparison categories, whereas differences for coordination complexity and motor skill learning demand were smaller, but no omnibus or direct moderator test was significant. All four moderator conclusions were unchanged across alternative sampling-covariance structures. For environmental predictability and cognitive engagement, covariate-adjusted models and analyses restricted to high-confidence coded arms were also consistent with the unadjusted results.

These findings should not be interpreted as evidence that qualitative exercise characteristics are irrelevant or equivalent. Most trials were not designed to isolate one characteristic while holding other intervention features constant, and programs could differ simultaneously in intensity, novelty, social interaction, instructor contact, adherence, feedback, and comparator matching. The four dimensions also overlapped conceptually and empirically; motor skill learning demand was particularly related to coordination complexity and was therefore treated as a supplementary exploratory moderator. Aggregate-data meta-regression cannot fully separate correlated features or establish an independent causal contribution of any single characteristic (Thompson and Higgins, 2002).

Measurement limitations further constrain interpretation. Skill characteristics were inferred from published intervention descriptions rather than measured directly during delivery, and reports varied in detail regarding task rules, progression, feedback, decision-making, environmental changes, and actual cognitive or motor load (Hoffmann et al., 2014; Slade et al., 2016). Coding confidence reflected reporting completeness and clarity but did not externally validate classifications or eliminate misclassification.

Reproducibility also warrants qualification. Two reviewers independently coded intervention arms and resolved disagreements by consensus, with third-reviewer consultation when needed. However, reviewer-specific pre-consensus files were not retained, so formal inter-rater agreement statistics could not be calculated retrospectively without a new coding exercise; no retrospective reliability coefficient was generated. During revision, operational definitions and decision rules were consolidated in a coding codebook to improve transparency and facilitate interpretation and replication. This revision-stage clarification documents the decision framework but does not provide a retrospective reliability estimate.

Analytical categorization introduced additional constraints. Cognitive engagement, coordination complexity, and motor skill learning demand were initially coded as low, moderate, or high, but low and moderate categories were combined *post hoc* because of sparse cells. This improved estimability but reduced granularity and may have obscured nonlinear or threshold relationships. Challenge-point accounts suggest that task difficulty may depend on the relation between task demands and individual capability (Guadagnoli and Lee, 2004; Pesce, 2012; Tomporowski et al., 2015), which arm-level categories cannot fully capture.

Broader classifications were also sparse: only two open-skill arms and two arms without skill progression were identified. The evidence therefore cannot establish equivalence or superiority among open-, mixed-, and closed-skill exercise or between programs with and without explicit progression. Overall, the current evidence does not identify the four coded characteristics as reliable moderators. This does not show that they are unimportant; rather, the available evidence and arm-level operationalization were insufficient to demonstrate reproducible differences. More informative tests will require trials that directly manipulate specific cognitive-motor demands while holding other intervention features as constant as feasible, together with prospective operational definitions and detailed reporting.

### 4.5 Additional moderator analyses, robustness, and certainty of interpretation

Intervention duration was not significantly associated with effect magnitude in either linear or duration-doubling models, including analyses restricted to 2–16 weeks. Interpretation remains cautious because only two trials exceeded 16 weeks, the duration distribution was uneven, and the full-range meta-regressions had very small Satterthwaite-adjusted denominator degrees of freedom. Thus, non-significant findings do not establish that duration is unimportant or that shorter and longer interventions are equivalent. Duration also captures only one component of exercise exposure and does not reflect frequency, intensity, session length, progression, adherence, or completed dose.

Comparator-specific point estimates differed numerically, but omnibus tests were not significant under either diagonal or non-diagonal covariance specifications. Because comparator type was not randomly assigned and covaried with population, setting, intervention content, attention, social contact, and study design, these contrasts are aggregate-level observational comparisons rather than causal evidence that passive controls inflate effects or active controls attenuate them (Thompson and Higgins, 2002). Well-matched active controls nevertheless remain important for separating exercise-specific effects from nonspecific influences.

The overall pooled mean was comparatively robust across alternative covariance structures, ICC assumptions, change-score specifications, baseline-imbalance exclusions, removal of high-risk trials, coding-confidence restrictions, comparator restrictions, exclusion of the trial requiring an assumed ICC, and leave-one-trial-out analyses. The largest overall leave-one-trial-out change was approximately 5.35%, reducing the likelihood that the positive mean reflected a single trial or analytical decision.

This overall stability should be distinguished from stage-specific precision. Leave-one-trial-out variation was greater in children and adolescents than in older adults, with maximum relative changes of approximately 18.46%, 20.79%, and 9.98%, respectively. The adolescent estimate was particularly sensitive because only nine trials contributed. Moreover, prediction intervals crossed zero under primary and alternative covariance assumptions. Sensitivity analyses therefore support robustness of the average direction but do not demonstrate homogeneous effects or uniform benefit across populations and settings.

Risk of bias remained important. No trial was rated at overall low risk, 44 had some concerns, and three were at high risk. Excluding high-risk trials produced a similar pooled estimate, but this cannot correct potential bias among trials with some concerns. Small-study-effect diagnostics were also inconsistent: sample-size-based and study-level analyses were non-significant, whereas standard-error-based multilevel models were significant and may have been affected by mathematical coupling (Pustejovsky and Rodgers, 2019). Publication bias therefore remained uncertain, particularly given restriction to English-language published journal articles and the absence of grey-literature searching.

Accordingly, certainty of evidence was rated as low for overall executive function and each executive-function domain, with downgrading for serious risk of bias and serious inconsistency. Statistical robustness and certainty address different questions: robustness indicates that the average estimate was not highly dependent on the analytical assumptions examined, whereas certainty reflects confidence in the underlying evidence base. A stable pooled mean therefore does not imply high certainty, low heterogeneity, or uniform applicability.

### 4.6 Methodological and practical implications

Methodologically, this review highlights the importance of explicitly addressing dependence in exercise–cognition meta-analysis. Trials often contributed multiple executive-function tasks, outcomes, intervention arms, and contrasts. Treating these estimates as independent may understate uncertainty, whereas selecting or averaging outcomes can discard information and make conclusions dependent on arbitrary aggregation rules. The three-level model retained eligible effect sizes while representing heterogeneity within and between trials (Van den Noortgate et al., 2013; Cheung, 2014; Assink and Wibbelink, 2016).

The dependency procedures addressed complementary statistical issues. The multilevel random-effects structure represented within- and between-trial heterogeneity; Study_ID-clustered CR2 inference provided small-sample-adjusted coefficient-level uncertainty; and non-diagonal variance–covariance matrices tested sensitivity to plausible within-sample correlations and shared-arm overlap (Tipton, 2015; Pustejovsky and Tipton, 2018). Stability across these specifications reduces concern that the main inferences were artifacts of the primary diagonal working structure, although these methods cannot eliminate unexplained heterogeneity or limitations in study design and reporting.

Clear distinctions among trials, independent participant samples, intervention arms, comparator arms, and effect-size estimates are also essential. Shared comparator groups should not be counted as independent samples merely because they contribute multiple contrasts, and actual participant counts should remain distinct from design-adjusted effective sample sizes used for variance calculations. More transparent arm-level reporting and identifiers for shared groups and repeated outcomes would improve reproducibility and interpretation.

Intervention reporting also requires greater detail. Broad modality labels may conceal differences in environmental predictability, cognitive engagement, coordination, progression, feedback, decision-making, and motor skill learning (Best, 2010; Pesce, 2012; Tomporowski et al., 2015). Future trials should report both conventional dose characteristics and recurring cognitive–motor demands, together with adherence and actual participation, to support replication and more reliable classification (Hoffmann et al., 2014; Slade et al., 2016).

Practically, structured exercise may be considered a potentially useful component of programs supporting executive-function performance, but the evidence does not establish superiority of cognitively demanding, coordinatively complex, open-skill, longer-duration, or other broadly defined exercise categories. Program selection should therefore consider safety, feasibility, accessibility, participant preference, adherence, physical-health objectives, available resources, and developmental or functional needs rather than assumed cognitive superiority of a particular exercise label.

Because prediction intervals crossed zero and certainty of evidence was low, the pooled mean should not be interpreted as guaranteeing benefit in every individual or setting. Where cognitive outcomes are relevant, participation, adherence, feasibility, adverse events, and cognitive performance should be monitored. Overall, the evidence supports structured exercise as a potentially beneficial but context-dependent component of broader health, educational, or functional programs.

### 4.7 Strengths and limitations

This review has several methodological strengths. It was restricted to randomized controlled trials and synthesized 202 effect-size estimates from 47 trials, 49 independent participant samples, and 4,195 actual participants. The three-level framework retained multiple eligible outcomes and contrasts while modeling heterogeneity within and between trials, and Study_ID-clustered CR2 inference provided small-sample-adjusted robust uncertainty estimates. Dependence assumptions were further examined using non-diagonal variance–covariance matrices incorporating plausible within-sample correlations and shared-arm structures. Robustness was assessed across covariance assumptions, cluster adjustments, effect-size specifications, baseline-imbalance exclusions, risk-of-bias and coding restrictions, comparator restrictions, and leave-one-trial-out analyses. The review also distinguished actual participant counts from design-adjusted effective sample sizes and characterized qualitative exercise features at the intervention-arm level rather than relying solely on modality labels.

Several limitations qualify interpretation. First, substantial unexplained heterogeneity remained under all covariance specifications, and prediction intervals crossed zero. Thus, the positive pooled mean does not imply uniform benefit. Potential sources of heterogeneity, including exercise intensity, frequency, adherence, supervision, baseline fitness, socioeconomic context, and task reliability, were inconsistently reported or not jointly modeled. Moreover, true within-sample sampling correlations were unknown; sensitivity analyses evaluate robustness to plausible assumptions but cannot identify the true covariance structure.

Second, risk-of-bias concerns were widespread. No trial was rated at overall low risk, 44 had some concerns, and three were at high risk. Excluding high-risk trials did not materially alter the pooled estimate, but this cannot correct potential bias among trials with some concerns. Statistical robustness should therefore not be equated with high certainty.

Third, moderator analyses were based on aggregate study-, sample-, arm-, comparator-, and outcome-level characteristics rather than individual-participant data or randomized moderator contrasts. They cannot establish independent causal effects of developmental stage, duration, comparator type, or skill characteristics (Thompson and Higgins, 2002), and non-significant tests do not establish equivalence. This is particularly relevant for adolescents, who were represented by only nine trials, 10 independent samples, and 756 participants; leave-one-trial-out analyses indicated greater uncertainty in effect magnitude than in older adults.

Fourth, the exercise skill-characteristic framework was review-specific and depended on published intervention descriptions. The four dimensions were inferred rather than measured directly, coding confidence did not provide external validation, and *post hoc* merging of low and moderate categories reduced granularity. Open-skill and no-progression categories were too sparse for formal inference, and overlap among dimensions limited simultaneous modeling. Although two reviewers independently completed the original coding, reviewer-specific pre-consensus files were not retained; formal inter-rater agreement could therefore not be calculated retrospectively without a new coding exercise. The revision-stage codebook improves transparency of the final decision rules but does not constitute a retrospective reliability assessment.

Fifth, intervention-duration evidence was limited. Only two trials exceeded 16 weeks, the distribution was uneven, and some meta-regressions had very small effective denominator degrees of freedom. Non-significant findings therefore do not establish equivalence between shorter and longer programs. Follow-up outcomes were not synthesized separately, so persistence of effects after intervention completion could not be evaluated systematically.

Sixth, the evidence base was intentionally restricted. Although five databases were searched from inception to May 20, 2026 without interface date or language filters, eligibility during screening was limited to formally published English-language journal articles dated from January 1, 2015 to May 20, 2026. The 2015 cutoff was a pragmatic scope restriction intended to focus on contemporary randomized evidence and should not be interpreted as implying that earlier trials were inferior or irrelevant. Nevertheless, excluding earlier studies reduces historical completeness. The English-language restriction may have introduced language bias, and exclusion of dissertations, conference proceedings, trial registries, and other grey literature may have increased vulnerability to publication bias. Reference-list and forward-citation searching were also not undertaken. These limitations are not resolved by the inconsistent small-study-effect diagnostics.

Seventh, the PROSPERO record was updated after initial registration. Versions 2.0 and 3.0 were retrospective relative to the initial synthesis, and some eligibility details, moderator definitions, and analytical procedures were introduced or materially refined thereafter. These changes are documented with timing and rationale in Supplementary Table S5. Study authors were also not contacted for missing or unclear information despite this having been planned initially.

Finally, executive-function tasks are not process-pure (Miyake et al., 2000; Karr et al., 2018). Domain assignment necessarily simplified multidimensional performance and may have introduced classification error. Young and middle-aged adults were outside the eligibility criteria, so developmental-stage findings should not be generalized to those populations.

### 4.8 Future research directions

Future trials should move beyond broad exercise labels and directly manipulate theoretically relevant cognitive and motor demands. Head-to-head randomized designs could vary environmental predictability, cognitive engagement, coordination complexity, or motor skill learning demand while matching physical dose, instructor contact, social interaction, expectancy, and comparator exposure as closely as feasible (Best, 2010; Pesce, 2012; Tomporowski et al., 2015; Diamond and Ling, 2016). Manipulation checks and fidelity measures should verify that intended cognitive–motor demands were actually delivered.

Intervention reporting should describe task rules, progression, novelty, feedback, decision-making demands, environmental changes, adherence, fidelity, and achieved exposure in addition to conventional dose variables (Hoffmann et al., 2014; Slade et al., 2016). Coding frameworks should be prespecified, reviewer-specific pre-consensus records retained, and inter-rater agreement reported where appropriate. External validation against participant- or intervention-level measures of cognitive and motor demand would further strengthen classification.

Developmental coverage also requires improvement. The adolescent evidence base comprised only nine trials, limiting precision. Larger, adequately powered trials using comparable interventions, comparators, and outcome measures across age groups are needed to test developmental moderation and domain-by-stage interactions more directly.

Executive-function assessment should use reliable, developmentally appropriate measures and avoid assuming that single tasks are process-pure (Miyake et al., 2000; Karr et al., 2018). Prospective registration should specify primary domains, tasks, metrics, time points, analysis populations, and statistical models, with deviations reported transparently (Higgins et al., 2024; Sterne et al., 2019).

Future studies should also improve reporting for dependency-aware synthesis by identifying shared participant groups and reporting repeated-measure correlations, cluster characteristics, intracluster correlations, and paired information where relevant (Morris, 2008; Van den Noortgate et al., 2013). Longer interventions and follow-up assessments are needed to examine persistence of effects and to evaluate duration alongside intensity, frequency, adherence, progression, and completed exposure. Individual-participant-data meta-analysis may further clarify effect modification by participant characteristics (Riley et al., 2010).

Finally, future reviews should broaden literature coverage to non-English publications, grey literature, trial registries, dissertations, conference proceedings, and citation searching while prospectively documenting eligibility and analytical decisions. Until more direct comparative evidence is available, claims that one exercise skill category or developmental stage produces larger executive-function benefits should remain tentative.

## 5 Conclusion

This systematic review and three-level meta-analysis synthesized 202 effect-size estimates from 47 randomized controlled trials representing 49 independent participant samples and 4,195 actual participants. Structured exercise was associated with a positive average effect on executive-function performance relative to eligible comparators (Hedges’ *g* = 0.412, Study_ID-clustered CR2 robust 95% CI [0.284, 0.540]), and this estimate was generally robust across alternative covariance assumptions and sensitivity analyses.

Positive pooled estimates were observed for inhibitory control, working memory, and cognitive flexibility and across childhood, adolescence, and later life. However, no statistically reliable moderation was identified for executive-function domain, developmental stage, environmental predictability, cognitive engagement, coordination complexity, motor skill learning demand, intervention duration, or comparator type. These non-significant findings do not demonstrate equivalence or establish that these characteristics are unimportant.

Interpretation remains cautious because substantial unexplained heterogeneity persisted and the primary prediction interval crossed zero. Stage-specific estimates were also less precise, particularly for adolescents, whose evidence base comprised only nine trials. Certainty of evidence was low because of serious risk of bias and inconsistency, and publication bias remained uncertain.

Overall, structured exercise may support executive-function performance on average, but the magnitude and consistency of benefit appear context dependent. Current evidence does not identify a developmental stage, executive-function domain, duration, comparator type, or broadly defined exercise skill characteristic associated with reliably larger effects. More direct, adequately powered, and prospectively specified comparative trials are needed to determine whether particular cognitive–motor demands meaningfully modify executive-function responses.

## Data Availability

The original contributions presented in the study are included in the article/Supplementary material, further inquiries can be directed to the corresponding author.
