# Supplementary material for: Effects of structured exercise on executive function across childhood, adolescence, and later life: a systematic review and three-level meta-analysis examining exercise skill characteristics
Source: Front Psychol. 2026 Oct 14;17:1952545. doi: 10.3389/fpsyg.2026.1952545 (PMC13713456; doi:10.3389/fpsyg.2026.1952545)
Supplement: Supplementary file 1 [file Supplementary_file_1.docx]

Supplementary Material

Supplementary Tables S1–S6

This supplementary file contains Supplementary Tables S1–S6. The accompanying Excel workbook is provided separately as Supplementary Dataset S1.

**Supplementary Table S1. Database-specific search strategies used to identify randomized controlled trials examining the effects of structured exercise on executive function across childhood, adolescence, and later life.**

| **Database** | **Search strategy** | **Records identified, n** |
| --- | --- | --- |
| PubMed | ( "Exercise"[Mesh] OR "Motor Activity"[Mesh] OR "Physical Fitness"[Mesh] OR exercise[tiab] OR exercises[tiab] OR "physical activity"[tiab] OR "physical activities"[tiab] OR "physical exercise"[tiab] OR "exercise training"[tiab] OR "physical training"[tiab] OR "aerobic exercise"[tiab] OR "aerobic training"[tiab] OR "resistance training"[tiab] OR "strength training"[tiab] OR "endurance training"[tiab] OR "balance training"[tiab] OR "coordination training"[tiab] OR "motor training"[tiab] OR "neuromotor training"[tiab] OR "tai chi"[tiab] OR taiji[tiab] OR qigong[tiab] OR yoga[tiab] OR dance[tiab] OR dancing[tiab] OR "dance training"[tiab] OR exergam*[tiab] OR "active video game*"[tiab] OR "video game training"[tiab] OR "virtual reality training"[tiab] OR "dual-task training"[tiab] OR "dual task training"[tiab] OR "cognitive-motor training"[tiab] OR "motor-cognitive training"[tiab] OR "open-skill"[tiab] OR "closed-skill"[tiab] OR "open skill"[tiab] OR "closed skill"[tiab] OR "table tennis"[tiab] OR badminton[tiab] OR tennis[tiab] OR basketball[tiab] OR soccer[tiab] OR football[tiab] OR volleyball[tiab] OR "martial art*"[tiab] ) AND ( "Executive Function"[Mesh] OR "Neuropsychological Tests"[Mesh] OR "executive function"[tiab] OR "executive functions"[tiab] OR "executive control"[tiab] OR "cognitive control"[tiab] OR "cognitive function"[tiab] OR "cognitive functions"[tiab] OR "cognitive performance"[tiab] OR "neurocognitive function"[tiab] OR "neurocognitive performance"[tiab] OR "neuropsychological performance"[tiab] OR "inhibitory control"[tiab] OR inhibition[tiab] OR "response inhibition"[tiab] OR "working memory"[tiab] OR "cognitive flexibility"[tiab] OR "mental flexibility"[tiab] OR "task switching"[tiab] OR "set shifting"[tiab] OR Stroop[tiab] OR Flanker[tiab] OR "go/no-go"[tiab] OR "go no go"[tiab] OR "n-back"[tiab] OR "Trail Making"[tiab] OR TMT[tiab] OR "Wisconsin Card Sorting"[tiab] OR WCST[tiab] OR "Digit Span"[tiab] ) AND ( "Child"[Mesh] OR "Adolescent"[Mesh] OR "Aged"[Mesh] OR "Aged, 80 and over"[Mesh] OR child*[tiab] OR preschool*[tiab] OR schoolchild*[tiab] OR "school-aged"[tiab] OR student*[tiab] OR school[tiab] OR classroom[tiab] OR adolescent*[tiab] OR teenager*[tiab] OR youth*[tiab] OR "young people"[tiab] OR "older adult*"[tiab] OR elderly[tiab] OR senior*[tiab] OR geriatric*[tiab] OR "community-dwelling"[tiab] ) AND ( randomized controlled trial[pt] OR controlled clinical trial[pt] OR random*[tiab] OR randomized[tiab] OR randomised[tiab] OR randomly[tiab] OR "randomized trial"[tiab] OR "randomised trial"[tiab] OR "controlled trial"[tiab] ) NOT ( animals[mh] NOT humans[mh] ) NOT ( review[pt] OR systematic review[pt] OR meta-analysis[pt] OR protocol[ti] ) | 3164 |
| Embase | ('exercise'/exp OR 'physical fitness'/exp OR exercise:ti,ab,kw OR exercises:ti,ab,kw OR 'physical activity':ti,ab,kw OR 'physical exercise':ti,ab,kw OR 'exercise training':ti,ab,kw OR 'physical training':ti,ab,kw OR 'aerobic exercise':ti,ab,kw OR 'aerobic training':ti,ab,kw OR 'resistance training':ti,ab,kw OR 'strength training':ti,ab,kw OR 'endurance training':ti,ab,kw OR 'balance training':ti,ab,kw OR 'coordination training':ti,ab,kw OR 'motor training':ti,ab,kw OR 'neuromotor training':ti,ab,kw OR 'tai chi':ti,ab,kw OR taiji:ti,ab,kw OR qigong:ti,ab,kw OR yoga:ti,ab,kw OR dance:ti,ab,kw OR dancing:ti,ab,kw OR 'dance training':ti,ab,kw OR exergam*:ti,ab,kw OR 'active video game*':ti,ab,kw OR 'video game training':ti,ab,kw OR 'virtual reality training':ti,ab,kw OR 'dual-task training':ti,ab,kw OR 'dual task training':ti,ab,kw OR 'cognitive-motor training':ti,ab,kw OR 'motor-cognitive training':ti,ab,kw OR 'open-skill':ti,ab,kw OR 'closed-skill':ti,ab,kw OR 'open skill':ti,ab,kw OR 'closed skill':ti,ab,kw OR 'table tennis':ti,ab,kw OR badminton:ti,ab,kw OR tennis:ti,ab,kw OR basketball:ti,ab,kw OR soccer:ti,ab,kw OR football:ti,ab,kw OR volleyball:ti,ab,kw OR 'martial art*':ti,ab,kw) AND ('executive function'/exp OR 'executive function':ti,ab,kw OR 'executive functions':ti,ab,kw OR 'executive control':ti,ab,kw OR 'cognitive control':ti,ab,kw OR 'cognitive function':ti,ab,kw OR 'cognitive functions':ti,ab,kw OR 'cognitive performance':ti,ab,kw OR 'neurocognitive function':ti,ab,kw OR 'neurocognitive performance':ti,ab,kw OR 'inhibitory control':ti,ab,kw OR inhibition:ti,ab,kw OR 'response inhibition':ti,ab,kw OR 'working memory':ti,ab,kw OR 'cognitive flexibility':ti,ab,kw OR 'mental flexibility':ti,ab,kw OR 'task switching':ti,ab,kw OR 'set shifting':ti,ab,kw OR stroop:ti,ab,kw OR flanker:ti,ab,kw OR 'go/no-go':ti,ab,kw OR 'go no go':ti,ab,kw OR 'n-back':ti,ab,kw OR 'trail making':ti,ab,kw OR tmt:ti,ab,kw OR 'wisconsin card sorting':ti,ab,kw OR wcst:ti,ab,kw OR 'digit span':ti,ab,kw) AND ('child'/exp OR 'adolescent'/exp OR 'aged'/exp OR child*:ti,ab,kw OR preschool*:ti,ab,kw OR schoolchild*:ti,ab,kw OR 'school-aged':ti,ab,kw OR adolescent*:ti,ab,kw OR teenager*:ti,ab,kw OR youth*:ti,ab,kw OR 'young people':ti,ab,kw OR 'older adult*':ti,ab,kw OR elderly:ti,ab,kw OR senior*:ti,ab,kw OR geriatric*:ti,ab,kw OR 'community-dwelling':ti,ab,kw) AND ('randomized controlled trial'/exp OR 'controlled clinical trial'/exp OR random*:ti,ab,kw OR randomized:ti,ab,kw OR randomised:ti,ab,kw OR randomly:ti,ab,kw OR 'randomized trial':ti,ab,kw OR 'randomised trial':ti,ab,kw OR 'controlled trial':ti,ab,kw) NOT ('animal'/exp NOT 'human'/exp) NOT ('review'/it OR 'systematic review'/it OR 'meta analysis'/it OR protocol:ti OR 'conference abstract'/it) | 4259 |
| Web of Science | TS=(exercise OR exercises OR "physical activity" OR "physical activities" OR "physical exercise" OR "exercise training" OR "physical training" OR "aerobic exercise" OR "aerobic training" OR "resistance training" OR "strength training" OR "endurance training" OR "balance training" OR "coordination training" OR "motor training" OR "neuromotor training" OR "tai chi" OR taiji OR qigong OR yoga OR dance OR dancing OR "dance training" OR exergam* OR "active video game*" OR "video game training" OR "virtual reality training" OR "dual-task training" OR "dual task training" OR "cognitive-motor training" OR "motor-cognitive training" OR "open-skill" OR "closed-skill" OR "open skill" OR "closed skill" OR "table tennis" OR badminton OR tennis OR basketball OR soccer OR football OR volleyball OR "martial art*") AND TS=("executive function" OR "executive functions" OR "executive control" OR "cognitive control" OR "cognitive function" OR "cognitive functions" OR "cognitive performance" OR "neurocognitive function" OR "neurocognitive performance" OR "neuropsychological performance" OR "inhibitory control" OR inhibition OR "response inhibition" OR "working memory" OR "cognitive flexibility" OR "mental flexibility" OR "task switching" OR "set shifting" OR Stroop OR Flanker OR "go/no-go" OR "go no go" OR "n-back" OR "Trail Making" OR TMT OR "Wisconsin Card Sorting" OR WCST OR "Digit Span") AND TS=(child* OR preschool* OR schoolchild* OR "school-aged" OR student* OR school OR classroom OR adolescent* OR teenager* OR youth* OR "young people" OR "older adult*" OR elderly OR senior* OR geriatric* OR "community-dwelling") AND TS=(random* OR randomized OR randomised OR randomly OR "randomized trial" OR "randomised trial" OR "controlled trial") NOT TS=("systematic review" OR "meta-analysis" OR "meta analysis" OR protocol) | 2803 |
| Scopus | TITLE-ABS-KEY ( exercise OR exercises OR "physical activity" OR "physical activities" OR "physical exercise" OR "exercise training" OR "physical training" OR "aerobic exercise" OR "aerobic training" OR "resistance training" OR "strength training" OR "endurance training" OR "balance training" OR "coordination training" OR "motor training" OR "neuromotor training" OR "tai chi" OR taiji OR qigong OR yoga OR dance OR dancing OR "dance training" OR exergam* OR "active video game*" OR "video game training" OR "virtual reality training" OR "dual-task training" OR "dual task training" OR "cognitive-motor training" OR "motor-cognitive training" OR "open-skill" OR "closed-skill" OR "open skill" OR "closed skill" OR "table tennis" OR badminton OR tennis OR basketball OR soccer OR football OR volleyball OR "martial art*" ) AND TITLE-ABS-KEY ( "executive function" OR "executive functions" OR "executive control" OR "cognitive control" OR "cognitive function" OR "cognitive functions" OR "cognitive performance" OR "neurocognitive function" OR "neurocognitive performance" OR "neuropsychological performance" OR "inhibitory control" OR inhibition OR "response inhibition" OR "working memory" OR "cognitive flexibility" OR "mental flexibility" OR "task switching" OR "set shifting" OR Stroop OR Flanker OR "go/no-go" OR "go no go" OR "n-back" OR "Trail Making" OR TMT OR "Wisconsin Card Sorting" OR WCST OR "Digit Span" ) AND TITLE-ABS-KEY ( child* OR preschool* OR schoolchild* OR "school-aged" OR student* OR school OR classroom OR adolescent* OR teenager* OR youth* OR "young people" OR "older adult*" OR elderly OR senior* OR geriatric* OR "community-dwelling" ) AND TITLE-ABS-KEY ( random* OR randomized OR randomised OR randomly OR "randomized trial" OR "randomised trial" OR "controlled trial" ) AND NOT TITLE-ABS-KEY ( "systematic review" OR "meta-analysis" OR "meta analysis" OR protocol ) | 2812 |
| Cochrane Library | #1(child* OR adolescen* OR teenager* OR teen* OR youth* OR "young people" OR "young person*" OR pediatric* OR paediatric* OR schoolchild* OR student* OR "primary school" OR "secondary school" OR "middle school" OR "high school" OR elder* OR elderly OR aged OR aging OR ageing OR "older adult*" OR "older people" OR "older person*" OR senior* OR geriatric* OR "late life" OR "later life"):ti,ab,kw  #2(exercise OR exercises OR "physical activity" OR "physical activities" OR "physical exercise" OR "exercise training" OR "physical training" OR "aerobic exercise" OR "aerobic training" OR "resistance training" OR "strength training" OR "endurance training" OR "balance training" OR "coordination training" OR "motor training" OR "neuromotor training" OR "tai chi" OR taiji OR qigong OR yoga OR dance OR dancing OR "dance training" OR exergam* OR active NEXT video NEXT game* OR "video game training" OR "virtual reality training" OR "dual-task training" OR "dual task training" OR "cognitive-motor training" OR "motor-cognitive training" OR "open-skill" OR "closed-skill" OR "open skill" OR "closed skill" OR "table tennis" OR badminton OR tennis OR basketball OR soccer OR football OR volleyball OR martial NEXT art*):ti,ab,kw  #3("executive function" OR "executive functions" OR "executive functioning" OR "executive control" OR "cognitive control" OR "inhibitory control" OR inhibition OR "response inhibition" OR "interference control" OR "working memory" OR "updating" OR "cognitive flexibility" OR flexibility OR "mental flexibility" OR "set shifting" OR set-shifting OR switching OR "task switching" OR "attention switching" OR planning OR "problem solving" OR Stroop OR Flanker OR "Simon task" OR "go/no-go" OR "go no go" OR "stop-signal" OR "stop signal" OR "n-back" OR nback OR "digit span" OR "Corsi" OR "trail making" OR "Trail Making Test" OR TMT OR WCST OR "Wisconsin Card Sorting Test"):ti,ab,kw  #4(random* OR trial OR "controlled trial" OR "clinical trial" OR RCT OR "randomized controlled trial" OR "randomised controlled trial" OR placebo OR "wait-list" OR waitlist OR "usual care" OR control OR "control group"):ti,ab,kw  #1 AND #2 AND #3 AND #4 | 5308 |

**Note.** Searches were conducted in PubMed, Embase, the Cochrane Library (CENTRAL), Scopus, and Web of Science from database inception to May 20, 2026. No publication-date or language restrictions were applied within the database interfaces. Eligibility was subsequently restricted during record screening to formally published English-language journal articles dated from January 1, 2015 to May 20, 2026. The 2015 eligibility cutoff was applied during screening rather than as a database search filter. Grey-literature sources, backward reference-list searching, and forward citation searching were not used. Search strategies were adapted to the syntax and indexing structure of each database.

**Supplementary Table S2. Final consensus exercise skill-characteristic coding and brief rationales for the eligible structured-exercise intervention arms.**

| **Study** | **Arm ID** | **Intervention arm / modality** | **Duration, weeks** | **Environmental predictability** | **Cognitive engagement** | **Coordination complexity** | **Motor skill learning demand** | **Coding confidence** | **Brief coding rationale** |
| --- | --- | --- | --- | --- | --- | --- | --- | --- | --- |
| Alexandrino et al. (2026) | NO.18_E1 | Short-duration HIIT group — HIIT running | 6 | Stable | Low-to-moderate | Low-to-moderate | Low-to-moderate | High | HIIT running in a stable setting; repetitive fitness movements with low-to-moderate cognitive, coordination, and motor-learning demands. |
| Antunes et al. (2015) | NO.138_E1 | Aerobic exercise — Cycling | 26 | Stable | Low-to-moderate | Low-to-moderate | Low-to-moderate | High | Cycling in a stable setting; repetitive fitness movement with low-to-moderate cognitive, coordination, and motor-learning demands. |
| Basarir et al. (2025) | NO.37_E1 | Coordination-based training group / Exercise group (EG) — Coordination-based, cognitively engaging movement | 8 | Semi-dynamic | High | High | High | High | Coordination-based, cognitively engaging movement with dual-task features; semi-dynamic setting and high cognitive, coordination, and motor-learning demands. |
| Cai et al. (2025) | NO.36_E1 | Classroom physical education exercise group — Soccer skills | 12 | Semi-dynamic | High | High | High | High | Basketball, soccer, ethnic dance, aerobics, and sport games combined structured practice with interactive content; semi-dynamic environment and high core skill demands. |
| Chen et al. (2016) | NO.125_E1 | Moderate PA program | 12 | Stable | Low-to-moderate | Low-to-moderate | Low-to-moderate | Moderate | Fast walking, stair climbing, rope skipping, and aerobic dance were participant-selected but predictable; low-to-moderate core skill demands and moderate confidence. |
| Cheng et al. (2023) | NO.60_E1 | Equine-assisted activity group (EAAG) — Physical activity | 12 | Semi-dynamic | High | High | High | Moderate | Externally paced horse movement required postural and coordination adjustments; semi-dynamic setting, high core skill demands, and moderate confidence from incomplete protocol reporting. |
| Cho et al. (2017) | NO.115_E1 | Taekwondo — Martial arts | 16 | Dynamic | High | High | High | High | Dynamic martial-arts sequences; high cognitive, coordination, and motor-learning demands. |
| Coetsee et al. (2017) | NO.114_E1 | High-intensity aerobic interval training (HIIT) — Aerobic+resistance | 16 | Stable | Low-to-moderate | Low-to-moderate | Low-to-moderate | High | Aerobic and resistance exercise in a stable setting; low-to-moderate cognitive, coordination, and motor-learning demands. |
| Coetsee et al. (2017) | NO.114_E2 | Moderate continuous aerobic training (MCT) — Aerobic+resistance | 16 | Stable | Low-to-moderate | Low-to-moderate | Low-to-moderate | High | Aerobic and resistance exercise in a stable setting; low-to-moderate cognitive, coordination, and motor-learning demands. |
| Coetsee et al. (2017) | NO.114_E3 | Resistance training (RT) | 16 | Stable | Low-to-moderate | Low-to-moderate | Low-to-moderate | High | Resistance exercise in a stable setting; predictable fitness movements with low-to-moderate cognitive, coordination, and motor-learning demands. |
| Cunha et al. (2026) | NO.16_E1 | 8–12RM RT — Resistance | 12 | Stable | Low-to-moderate | Low-to-moderate | Low-to-moderate | High | Resistance exercise in a stable setting; predictable fitness movements with low-to-moderate cognitive, coordination, and motor-learning demands. |
| Cunha et al. (2026) | NO.16_E2 | 10–15RM RT — Resistance | 12 | Stable | Low-to-moderate | Low-to-moderate | Low-to-moderate | High | Resistance exercise in a stable setting; predictable fitness movements with low-to-moderate cognitive, coordination, and motor-learning demands. |
| de Greeff et al. (2016) | NO.123_E1 | Physically active academic lessons (F&V) — Active lessons | 44 | Stable | High | Low-to-moderate | Low-to-moderate | Moderate | Predictable movements integrated with mathematics and language tasks; stable setting, high cognitive engagement, low-to-moderate coordination and motor-learning demands, and moderate confidence. |
| García-Garro et al. (2020) | NO.88_E1 | Pilates group | 12 | Stable | Low-to-moderate | Low-to-moderate | Low-to-moderate | High | Pilates sequences in a stable setting; predictable movements with low-to-moderate cognitive, coordination, and motor-learning demands. |
| Goumopoulos et al. (2026) | NO.14_E1 | GAME2AWE exergaming intervention — Exergame | 16 | Dynamic | High | High | High | High | Dynamic virtual gameplay and feedback; high cognitive, coordination, and motor-learning demands. |
| Hola et al. (2024) | NO.48_E1 | Dance group (DG) | 12 | Semi-dynamic | High | High | High | High | Dance progressed from imitation and fixed pathways to controlled improvisation; semi-dynamic setting and high cognitive, coordination, and motor-learning demands. |
| Hola et al. (2024) | NO.48_E2 | Martial arts group (MaG) — Tai Chi | 12 | Semi-dynamic | High | High | High | High | Fixed martial-arts techniques, shadow work, pads, bags, pair work, and model situations; semi-dynamic setting with high core skill demands. |
| Intawachirarat et al. (2026) | NO.12_E1 | Contemporary dance | 10 | Semi-dynamic | High | High | High | High | Dance rhythm and movement sequences in a semi-dynamic setting; high cognitive, coordination, and motor-learning demands. |
| Iuliano et al. (2015) | NO.135_E1 | CVG — Aerobic+resistance | 12 | Stable | Low-to-moderate | Low-to-moderate | Low-to-moderate | High | Aerobic and resistance exercise in a stable setting; low-to-moderate cognitive, coordination, and motor-learning demands. |
| Iuliano et al. (2015) | NO.135_E2 | RG — Resistance | 12 | Stable | Low-to-moderate | Low-to-moderate | Low-to-moderate | High | Resistance exercise in a stable setting; predictable fitness movements with low-to-moderate cognitive, coordination, and motor-learning demands. |
| Iuliano et al. (2015) | NO.135_E3 | PG — Balance/postural | 12 | Stable | Low-to-moderate | Low-to-moderate | Low-to-moderate | High | Flexibility, balance, core stability, relaxation, and breathing exercises in a stable, predictable setting; low-to-moderate cognitive, coordination, and motor-learning demands. |
| Kaseb et al. (2026) | NO.11_E1 | Resistance training (RT) | 8 | Stable | Low-to-moderate | Low-to-moderate | Low-to-moderate | High | Resistance exercise in a stable setting; predictable fitness movements with low-to-moderate cognitive, coordination, and motor-learning demands. |
| Kaseb et al. (2026) | NO.11_E2 | Resistance training with blood flow restriction (RT-BFR) | 8 | Stable | Low-to-moderate | Low-to-moderate | Low-to-moderate | High | Resistance exercise in a stable setting; predictable fitness movements with low-to-moderate cognitive, coordination, and motor-learning demands. |
| Kim et al. (2015) | NO.134_E1 | CET — Resistance | 16 | Stable | Low-to-moderate | Low-to-moderate | Low-to-moderate | High | Resistance exercise in a stable setting; predictable fitness movements with low-to-moderate cognitive, coordination, and motor-learning demands. |
| Ladawan et al. (2022) | NO.71_E1 | 4-movement Qigong exercise — Qigong / traditional mind-body | 8 | Stable | Low-to-moderate | Low-to-moderate | Low-to-moderate | High | Qigong in a stable setting; predictable traditional mind-body sequences with low-to-moderate cognitive, coordination, and motor-learning demands. |
| Liu et al. (2018) | NO.106_E1 | Coordination exercise / jump-rope training — Rope/circuit | 12 | Dynamic | High | High | High | High | Dynamic rope and circuit tasks with object manipulation; high cognitive, coordination, and motor-learning demands. |
| Liu et al. (2025) | NO.32_E1 | DanceSport intervention, 4–5 years | 8 | Semi-dynamic | High | High | High | High | Dance rhythm and movement sequences in a semi-dynamic setting; high cognitive, coordination, and motor-learning demands. |
| Liu et al. (2025) | NO.32_E2 | DanceSport intervention, 5–6 years | 8 | Semi-dynamic | High | High | High | High | Dance rhythm and movement sequences in a semi-dynamic setting; high cognitive, coordination, and motor-learning demands. |
| Liu et al. (2025) | NO.31_E1 | High-intensity interval rope skipping (HIIT) — Rope/circuit | 8 | Stable | High | High | High | High | Stable rope and circuit tasks with object manipulation; high cognitive, coordination, and motor-learning demands. |
| Liu et al. (2025) | NO.31_E2 | Moderate-intensity continuous rope skipping (MICT) — Rope/circuit | 8 | Stable | Low-to-moderate | High | High | High | Stable rope and circuit tasks with object manipulation; low-to-moderate cognitive engagement but high coordination and motor-learning demands. |
| Lu et al. (2016) | NO.118_E1 | 12-form Tai Chi | 16 | Stable | High | High | High | High | Tai Chi sequences in a stable setting; high cognitive, coordination, and motor-learning demands. |
| Ludyga et al. (2019) | NO.98_E1 | Aerobic training — HIIT running | 10 | Stable | Low-to-moderate | Low-to-moderate | Low-to-moderate | High | HIIT running in a stable setting; repetitive fitness movements with low-to-moderate cognitive, coordination, and motor-learning demands. |
| Ludyga et al. (2019) | NO.98_E2 | Coordinative training — Coordinative / motor-skill training | 10 | Stable | High | High | High | High | Stable coordinative and motor-skill tasks with dual-task features; high cognitive, coordination, and motor-learning demands. |
| Ludyga et al. (2018) | NO.105_E1 | Combined aerobic and coordinative school-break exercise — Combined aerobic + coordinative | 8 | Stable | High | High | Low-to-moderate | High | Stable combined aerobic and coordinative training; high cognitive and coordination demands with low-to-moderate motor-learning demand. |
| Martín-Martínez et al. (2015) | NO.133_E1 | Small-sided games — Team games | 8 | Dynamic | High | High | Low-to-moderate | High | Three-versus-three football, basketball, and handball required continuous responses to opponents, teammates, and ball movement; dynamic setting, high cognition and coordination, low-to-moderate motor-learning demand. |
| Meijer et al. (2021) | NO.80_E1 | Aerobic exercise intervention — Aerobic PE / circuit, | 14 | Stable | High | Low-to-moderate | Low-to-moderate | High | Stable aerobic physical education and circuit activities; high cognitive engagement with low-to-moderate coordination and motor-learning demands. |
| Meijer et al. (2021) | NO.80_E2 | Cognitively demanding exercise — Team games | 14 | Dynamic | High | High | High | High | Adapted dodgeball, basketball, and soccer required strategy, cooperation, anticipation, changing demands, and complex rules; dynamic setting with high core skill demands. |
| Milot et al. (2026) | NO.9_E1 | Physical training (PT) group — Resistance | 12 | Stable | Low-to-moderate | Low-to-moderate | Low-to-moderate | High | Preplanned stepper and strength exercises without opponent or environmental adaptation; stable setting and low-to-moderate core skill demands. |
| Moghadam et al. (2025) | NO.29_E1 | Body percussion rhythmic exercise group — Rhythmic body percussion | 12 | Semi-dynamic | High | High | High | High | Rhythmic body-percussion sequences in a semi-dynamic setting; dual-task features and high cognitive, coordination, and motor-learning demands. |
| Moreau et al. (2017) | NO.113_E1 | High-intensity training (HIT) — High-intensity interval/whole-body exercise training | 6 | Dynamic | Low-to-moderate | High | Low-to-moderate | High | Dynamic whole-body interval training with object-related tasks; low-to-moderate cognitive engagement, high coordination complexity, and low-to-moderate motor-learning demand. |
| Nishiguchi et al. (2015) | NO.132_E1 | Physical+cognitive exercise — Resistance | 12 | Stable | High | Low-to-moderate | Low-to-moderate | High | Stable resistance training; high cognitive engagement with low-to-moderate coordination and motor-learning demands. |
| Pacheco Lopes Filho et al. (2019) | NO.95_E1 | Karate Group — Martial arts | 12 | Semi-dynamic | High | High | High | High | Fixed kihon and kata combined with kumite and pair applications; semi-dynamic setting with high cognitive, coordination, and motor-learning demands. |
| Park et al. (2022) | NO.67_E1 | Moderate-intensity aerobic stepping exercise training — Bench stepping / aerobic | 5 | Stable | Low-to-moderate | Low-to-moderate | Low-to-moderate | High | Bench stepping and aerobic exercise in a stable setting; low-to-moderate cognitive, coordination, and motor-learning demands. |
| Purohit et al. (2017) | NO.112_E1 | Yoga program | 12 | Stable | High | Low-to-moderate | Low-to-moderate | High | Yoga sequences in a stable setting; high cognitive engagement with low-to-moderate coordination and motor-learning demands. |
| Roldan et al. (2025) | NO.28_E1 | Low-velocity resistance training (LVRT) | 8 | Stable | Low-to-moderate | Low-to-moderate | Low-to-moderate | High | Resistance exercise in a stable setting; predictable fitness movements with low-to-moderate cognitive, coordination, and motor-learning demands. |
| Roldan et al. (2025) | NO.28_E2 | High-velocity resistance training (HVRT) | 8 | Stable | Low-to-moderate | Low-to-moderate | Low-to-moderate | High | Resistance exercise in a stable setting; predictable fitness movements with low-to-moderate cognitive, coordination, and motor-learning demands. |
| Solianik et al. (2021) | NO.77_E1 | 8-form Yang-style Tai Chi | 10 | Stable | High | High | Low-to-moderate | High | Eight-form Yang-style Tai Chi was delivered in a stable, instructor-led group setting. The intervention involved coordinated multisegmental movements and sustained attention to posture and movement execution, supporting high cognitive and coordination demands; motor-skill learning demand was coded as low-to-moderate. |
| Stenman et al. (2017) | NO.111_E1 | High-intensity interval training (HIT) — Running HIIT plus modified | 2 | Stable | Low-to-moderate | Low-to-moderate | Low-to-moderate | High | Running HIIT in a stable setting; repetitive fitness movements with low-to-moderate cognitive, coordination, and motor-learning demands. |
| Wang et al. (2026) | NO.6_E1 | HIIE group — HIIT/circuit | 12 | Stable | Low-to-moderate | Low-to-moderate | Low-to-moderate | Moderate | Preplanned sprinting, rope skipping, and burpees in a stable circuit; no opponent-based adaptation; low-to-moderate core skill demands. |
| Wang et al. (2026) | NO.6_E2 | MICE group — Jogging | 12 | Stable | Low-to-moderate | Low-to-moderate | Low-to-moderate | High | Jogging in a stable setting; repetitive fitness movements with low-to-moderate cognitive, coordination, and motor-learning demands. |
| Wen et al. (2018) | NO.102_E1 | Mini-trampoline physical activity — Mini-trampoline / trampoline PA | 10 | Stable | Low-to-moderate | High | High | High | Stable mini-trampoline activities with object-related tasks; low-to-moderate cognitive engagement but high coordination and motor-learning demands. |
| Williams et al. (2022) | NO.65_E1 | School-based sprint training — HIIT/circuit | 2 | Stable | Low-to-moderate | Low-to-moderate | Low-to-moderate | High | HIIT and circuit exercise in a stable setting; repetitive fitness movements with low-to-moderate cognitive, coordination, and motor-learning demands. |
| Yang et al. (2020) | NO.85_E1 | Tai Chi Chuan group | 8 | Stable | High | Low-to-moderate | Low-to-moderate | High | Tai Chi sequences in a stable setting; high cognitive engagement with low-to-moderate coordination and motor-learning demands. |
| Yu et al. (2025) | NO.20_E1 | Exergame group | 10 | Dynamic | High | High | High | High | Dynamic virtual gameplay and feedback; high cognitive, coordination, and motor-learning demands. |
| Zhang et al. (2024) | NO.41_E1 | Moderate-intensity recreational gymnastics — Recreational gymnastics / coordinated | 12 | Semi-dynamic | High | High | High | High | Coordinated recreational gymnastics with dual-task elements; semi-dynamic setting and high cognitive, coordination, and motor-learning demands. |
| Zhang et al. (2026) | NO.1_E1 | Adapted tango dance group | 12 | Semi-dynamic | High | High | High | High | Tango rhythm and partner-oriented movement in a semi-dynamic setting; high cognitive, coordination, and motor-learning demands. |
| Zhao et al. (2022) | NO.63_E1 | Active video game group (IG) — Exergame | 12 | Dynamic | High | High | High | High | Dance, boxing, and virtual tennis used real-time feedback, scoring, ranking, and progression; dynamic setting with high cognitive, coordination, and motor-learning demands. |
| Zhao et al. (2022) | NO.62_E1 | Exergame training group (ET) | 12 | Dynamic | High | High | High | High | Fitness Boxing, Zumba, and virtual tennis used visual feedback and game rules; dynamic setting with high cognitive, coordination, and motor-learning demands. |
| Zhao et al. (2022) | NO.62_E2 | Aerobic dancing training group (ADT) — Dance | 12 | Stable | High | High | High | High | Dance routines in a stable setting; rhythm and sequence demands produced high cognitive, coordination, and motor-learning requirements. |
| Zhong et al. (2024) | NO.39_E1 | Volleyball physical training group — Volleyball training | 12 | Semi-dynamic | High | High | High | High | Structured volleyball drills plus group games and competitive scenarios; semi-dynamic setting with high cognitive, coordination, and motor-learning demands. |
| Zinelabidine et al. (2022) | NO.61_E1 | Aerobic dance experimental group (EG) | 8 | Stable | High | High | High | High | Aerobic-dance routines were learned, memorized, repeated, and performed to music predictably; high cognitive, coordination, and motor-learning demands. |

Note. Each row represents one eligible structured-exercise intervention arm; distinct intervention arms from multi-arm trials are presented separately. The classifications shown are the final consensus codes. Exercise skill characteristics were coded according to the dominant and recurring environmental, cognitive, coordinative, and motor-learning demands of the intervention program as a whole rather than the modality label alone. Environmental predictability was classified as stable, semi-dynamic, or dynamic. Cognitive engagement, coordination complexity, and motor skill learning demand were initially coded as low, moderate, or high; the low and moderate categories were combined post hoc as low-to-moderate for meta-regression because of sparse cells. Coding confidence was rated as high or moderate according to the completeness, clarity, and internal consistency of the available intervention descriptions. Brief rationales summarize the reported intervention features supporting each final classification using information from the main article and, where available, supplementary materials, protocols, or trial registrations. Two reviewers independently completed the original arm-level coding and resolved disagreements by consensus or third-reviewer consultation; however, the reviewer-specific pre-consensus coding files were not retained. Formal inter-rater agreement therefore could not be calculated retrospectively without conducting a new coding exercise. The accompanying revision-stage coding codebook in Supplementary Dataset S1 consolidates and clarifies the operational definitions and decision rules used to interpret the final consensus classifications; it does not constitute a new recoding exercise or a retrospective reliability assessment. Arm ID, unique intervention-arm identifier.

**Supplementary Table S3. Full-range and restricted-range intervention-duration meta-regression analyses.**

| **Analysis range** | **Duration specification** | **Sampling covariance structure** | **Data retained, n** | **Duration range, weeks** | **Duration slope, Δg** | **Robust 95% CI** | **Robust SE** | **Satterthwaite df** | **p value** |
| --- | --- | --- | --- | --- | --- | --- | --- | --- | --- |
| Full range | Linear, per additional 4 weeks | Diagonal sampling variances | 202 effect sizes / 47 trials | 2–44 | −0.034 | [−0.168, 0.099] | 0.033 | 2.14 | 0.401 |
| Full range | Linear, per additional 4 weeks | Non-diagonal, ρ = 0.50 | 202 effect sizes / 47 trials | 2–44 | −0.037 | [−0.166, 0.093] | 0.030 | 1.98 | 0.344 |
| Full range | Log2, per doubling of duration | Diagonal sampling variances | 202 effect sizes / 47 trials | 2–44 | −0.031 | [−0.245, 0.184] | 0.086 | 5.56 | 0.734 |
| Full range | Log2, per doubling of duration | Non-diagonal, ρ = 0.50 | 202 effect sizes / 47 trials | 2–44 | −0.046 | [−0.258, 0.165] | 0.086 | 6.03 | 0.611 |
| Restricted range | Linear, per additional 4 weeks | Diagonal sampling variances | 195 effect sizes / 45 trials | 2–16 | −0.059 | [−0.234, 0.116] | 0.081 | 12.66 | 0.481 |
| Restricted range | Linear, per additional 4 weeks | Non-diagonal, ρ = 0.50 | 195 effect sizes / 45 trials | 2–16 | −0.064 | [−0.235, 0.107] | 0.080 | 14.68 | 0.435 |
| Restricted range | Log2, per doubling of duration | Diagonal sampling variances | 195 effect sizes / 45 trials | 2–16 | −0.014 | [−0.262, 0.233] | 0.094 | 4.62 | 0.884 |
| Restricted range | Log2, per doubling of duration | Non-diagonal, ρ = 0.50 | 195 effect sizes / 45 trials | 2–16 | −0.028 | [−0.262, 0.207] | 0.096 | 6.01 | 0.784 |

Note. Duration was centered at 12 weeks. In the linear models, the regression coefficient represents the estimated change in Hedges’ *g* for each additional four weeks of intervention. In the base-2 logarithmic models, the coefficient represents the estimated change in Hedges’ *g* associated with a doubling of intervention duration. Robust inference used Study_ID-clustered CR2 variance estimation with Satterthwaite-adjusted degrees of freedom. Non-diagonal models assumed a within-sample sampling-error correlation of ρ = 0.50. The restricted-range analyses excluded the two trials with intervention durations of 26 and 44 weeks, leaving interventions ranging from 2 to 16 weeks. Negative Δg values indicate lower estimated Hedges’ *g* values with increasing intervention duration and should not be interpreted as evidence that longer interventions are harmful or inferior. CI, confidence interval; CR2, small-sample-corrected cluster-robust variance estimation.

**Supplementary Table S4. Sensitivity and robustness analyses of the overall effect, moderator inferences, and developmental-stage estimates.**

**Panel A. Overall pooled effect and heterogeneity under alternative sampling-covariance assumptions**

| **Sampling-covariance structure** | **Hedges' g (95% CI)** | **df** | **p** | **Variance components (study / effect)** | **Typical sampling variance** | **I² (%) (study / effect / total)** | **95% prediction interval** | **Relative change from diagonal (%)** |
| --- | --- | --- | --- | --- | --- | --- | --- | --- |
| Diagonal primary | 0.412 [0.284, 0.540] | 43.45 | <0.001 | 0.1324 / 0.0580 | 0.0653 | 51.79 / 22.66 / 74.45 | [-0.476, 1.299] | 0.00 |
| ρ = 0.30 | 0.405 [0.278, 0.531] | 42.78 | <0.001 | 0.1063 / 0.0808 | 0.0538 | 44.12 / 33.54 / 77.66 | [-0.475, 1.284] | -1.80 |
| ρ = 0.50 | 0.400 [0.273, 0.526] | 42.03 | <0.001 | 0.0908 / 0.0990 | 0.0408 | 39.37 / 42.94 / 82.31 | [-0.486, 1.285] | -3.01 |
| ρ = 0.70 | 0.395 [0.267, 0.522] | 41.09 | <0.001 | 0.0768 / 0.1197 | 0.0256 | 34.56 / 53.90 / 88.46 | [-0.507, 1.296] | -4.20 |

**Panel B. Sensitivity of moderator inferences to alternative sampling-covariance assumptions**

| **Moderator** | **Sampling-covariance structure** | **F** | **df1** | **df2** | **p** | **Conclusion changed vs diagonal?** |
| --- | --- | --- | --- | --- | --- | --- |
| Executive-function domain | Diagonal | 3.338 | 2 | 16.82 | 0.060 | No |
| Executive-function domain | ρ = 0.30 | 2.974 | 2 | 17.92 | 0.077 | No |
| Executive-function domain | ρ = 0.50 | 2.732 | 2 | 18.32 | 0.092 | No |
| Executive-function domain | ρ = 0.70 | 2.533 | 2 | 18.37 | 0.107 | No |
| Developmental stage | Diagonal | 0.195 | 2 | 21.92 | 0.825 | No |
| Developmental stage | ρ = 0.30 | 0.278 | 2 | 21.51 | 0.760 | No |
| Developmental stage | ρ = 0.50 | 0.335 | 2 | 21.14 | 0.719 | No |
| Developmental stage | ρ = 0.70 | 0.388 | 2 | 20.71 | 0.683 | No |
| Environmental predictability | Diagonal | 1.121 | 2 | 7.56 | 0.375 | No |
| Environmental predictability | ρ = 0.30 | 1.273 | 2 | 9.80 | 0.323 | No |
| Environmental predictability | ρ = 0.50 | 1.393 | 2 | 11.06 | 0.289 | No |
| Environmental predictability | ρ = 0.70 | 1.522 | 2 | 12.00 | 0.258 | No |
| Cognitive engagement | Diagonal | 0.525 | 1 | 31.61 | 0.474 | No |
| Cognitive engagement | ρ = 0.30 | 0.657 | 1 | 32.72 | 0.423 | No |
| Cognitive engagement | ρ = 0.50 | 0.762 | 1 | 33.03 | 0.389 | No |
| Cognitive engagement | ρ = 0.70 | 0.876 | 1 | 33.02 | 0.356 | No |
| Coordination complexity | Diagonal | 0.085 | 1 | 7.42 | 0.779 | No |
| Coordination complexity | ρ = 0.30 | 0.190 | 1 | 11.65 | 0.671 | No |
| Coordination complexity | ρ = 0.50 | 0.295 | 1 | 14.72 | 0.595 | No |
| Coordination complexity | ρ = 0.70 | 0.432 | 1 | 17.69 | 0.520 | No |
| Motor skill learning demand | Diagonal | 0.315 | 1 | 7.65 | 0.591 | No |
| Motor skill learning demand | ρ = 0.30 | 0.482 | 1 | 12.14 | 0.501 | No |
| Motor skill learning demand | ρ = 0.50 | 0.627 | 1 | 15.42 | 0.441 | No |
| Motor skill learning demand | ρ = 0.70 | 0.796 | 1 | 18.61 | 0.384 | No |

**Panel C. Developmental-stage-specific pooled effects under alternative sampling-covariance assumptions**

| **Developmental stage** | **Sampling-covariance structure** | **Hedges' g (95% CI)** | **df** | **p** | **Variance components (study / effect)** | **Typical sampling variance** | **Total I² (%)** | **95% prediction interval** | **Relative change from stage diagonal (%)** |
| --- | --- | --- | --- | --- | --- | --- | --- | --- | --- |
| Children | Diagonal | 0.368 [0.123, 0.613] | 15.12 | 0.005879 | 0.1609 / 0.1166 | 0.0503 | 84.65 | [-0.774, 1.511] | 0.00 |
| Children | ρ = 0.30 | 0.356 [0.113, 0.599] | 14.84 | 0.007043 | 0.1322 / 0.1441 | 0.0402 | 87.31 | [-0.784, 1.496] | -3.40 |
| Children | ρ = 0.50 | 0.348 [0.104, 0.592] | 14.56 | 0.008337 | 0.1170 / 0.1659 | 0.0298 | 90.47 | [-0.805, 1.501] | -5.53 |
| Children | ρ = 0.70 | 0.341 [0.095, 0.587] | 14.23 | 0.010103 | 0.1042 / 0.1901 | 0.0183 | 94.14 | [-0.834, 1.516] | -7.52 |
| Adolescents | Diagonal | 0.460 [0.221, 0.699] | 7.00 | 0.002649 | 0.0542 / 0.0000 | 0.0652 | 45.41 | [-0.125, 1.045] | 0.00 |
| Adolescents | ρ = 0.30 | 0.458 [0.222, 0.695] | 6.86 | 0.002623 | 0.0421 / 0.0000 | 0.0545 | 43.58 | [-0.067, 0.984] | -0.37 |
| Adolescents | ρ = 0.50 | 0.457 [0.221, 0.694] | 6.61 | 0.002784 | 0.0316 / 0.0094 | 0.0421 | 49.32 | [-0.062, 0.977] | -0.52 |
| Adolescents | ρ = 0.70 | 0.458 [0.220, 0.695] | 6.25 | 0.003111 | 0.0200 / 0.0242 | 0.0270 | 62.10 | [-0.077, 0.993] | -0.50 |
| Older adults | Diagonal | 0.443 [0.226, 0.659] | 19.35 | 0.000399 | 0.1770 / 0.0222 | 0.0988 | 66.85 | [-0.513, 1.398] | 0.00 |
| Older adults | ρ = 0.30 | 0.440 [0.224, 0.656] | 19.34 | 0.000408 | 0.1520 / 0.0402 | 0.0841 | 69.56 | [-0.499, 1.380] | -0.54 |
| Older adults | ρ = 0.50 | 0.438 [0.223, 0.654] | 19.28 | 0.000416 | 0.1340 / 0.0552 | 0.0658 | 74.19 | [-0.494, 1.371] | -1.01 |
| Older adults | ρ = 0.70 | 0.436 [0.221, 0.650] | 19.17 | 0.000425 | 0.1152 / 0.0722 | 0.0428 | 81.40 | [-0.492, 1.364] | -1.57 |

**Panel D. Overall and developmental-stage-specific leave-one-trial-out robustness summaries**

| **Analysis set** | **Models fitted / successful** | **Full-model g** | **LOTO g range** | **Models with 95% CI including 0** | **Most influential removed trial** | **g after removal** | **Maximum relative change (%)** |
| --- | --- | --- | --- | --- | --- | --- | --- |
| Overall | 47 / 47 | 0.412 | 0.390–0.426 | 0 | NO.32 | 0.390 | 5.35 |
| Children | 17 / 17 | 0.368 | 0.300–0.406 | 0 | NO.32 | 0.300 | 18.46 |
| Adolescents | 9 / 9 | 0.460 | 0.398–0.555 | 0 | NO.18 | 0.555 | 20.79 |
| Older adults | 21 / 21 | 0.443 | 0.398–0.475 | 0 | NO.85 | 0.398 | 9.98 |

**Panel E. Additional pooled-effect sensitivity analyses**

| **Sensitivity domain** | **Scenario** | **Data retained, n** | **Hedges' g (95% CI)** | **p** | **Change from primary, Δg** |
| --- | --- | --- | --- | --- | --- |
| Dependence assumptions | Cluster-trial ICC = 0.01 | 202 effects / 47 trials / 49 samples | 0.412 [0.285, 0.539] | <0.001 | 0.000 |
| Dependence assumptions | Cluster-trial ICC = 0.05 | 202 effects / 47 trials / 49 samples | 0.412 [0.285, 0.540] | <0.001 | 0.000 |
| Dependence assumptions | Cluster-trial ICC = 0.10 | 202 effects / 47 trials / 49 samples | 0.412 [0.285, 0.540] | <0.001 | 0.001 |
| Effect-size construction and baseline imbalance | Morris change-score effect, r = 0.30 | 202 effects / 47 trials / 49 samples | 0.426 [0.313, 0.540] | <0.001 | 0.014 |
| Effect-size construction and baseline imbalance | Morris change-score effect, r = 0.50 | 202 effects / 47 trials / 49 samples | 0.426 [0.313, 0.539] | <0.001 | 0.014 |
| Effect-size construction and baseline imbalance | Morris change-score effect, r = 0.70 | 202 effects / 47 trials / 49 samples | 0.425 [0.312, 0.538] | <0.001 | 0.013 |
| Effect-size construction and baseline imbalance | Post-test g after excluding \|baseline d\| ≥ 0.80 | 198 effects / 47 trials / 49 samples | 0.415 [0.287, 0.542] | <0.001 | 0.003 |
| Effect-size construction and baseline imbalance | Post-test g after excluding \|baseline d\| ≥ 0.50 | 176 effects / 46 trials / 48 samples | 0.446 [0.310, 0.582] | <0.001 | 0.034 |
| Study and coding restrictions | Exclude three trials at overall high risk of bias | 196 effects / 44 trials / 46 samples | 0.427 [0.293, 0.560] | <0.001 | 0.015 |
| Study and coding restrictions | High-confidence skill coding only | 186 effects / 44 trials / 46 samples | 0.426 [0.290, 0.561] | <0.001 | 0.014 |
| Study and coding restrictions | Passive or usual-activity comparators only | 116 effects / 29 trials / 29 samples | 0.457 [0.281, 0.634] | <0.001 | 0.045 |
| Study and coding restrictions | Exclude NO.105 with externally imputed ICC | 200 effects / 46 trials / 48 samples | 0.411 [0.281, 0.540] | <0.001 | -0.001 |

Note. Positive Hedges’ *g* values favor structured exercise. CR2 inference was clustered by Study_ID with Satterthwaite-adjusted degrees of freedom. The primary sampling-variance matrix was diagonal; alternative covariance models assumed within-sample correlations of ρ = 0.30, 0.50, and 0.70. In Panel A, changes in multilevel *I*² should be interpreted jointly with the absolute heterogeneity variance components and scenario-specific typical sampling variance. Panel B summarizes covariance sensitivity of the six moderator tests; none changed statistical-significance status. Panel C presents separate intercept-only developmental-stage models rather than the developmental-stage meta-regression; all stage-specific 95% prediction intervals crossed zero. Panel D summarizes leave-one-trial-out analyses removing one complete Study_ID at each iteration; the minimum Satterthwaite-adjusted degrees of freedom in the adolescent analyses was 4.73. Panel E presents 12 additional pooled-effect sensitivity scenarios; together with the three alternative covariance scenarios in Panel A, these comprise the 15 pooled-effect sensitivity scenarios shown in Figure 8. CI, confidence interval; CR2, small-sample-corrected cluster-robust variance estimation; ICC, intracluster correlation coefficient; PI, prediction interval.

**Supplementary Table S5. Major amendments and clarifications to the PROSPERO record across Versions 1.0–3.0**

| **Domain** | **Version 1.0** | **Version 2.0** | **Version 3.0 / final review method** | **Nature, reason, and effect of change** |
| --- | --- | --- | --- | --- |
| 1. Review scope and population | Focused on whether open- versus closed-skill exercise benefited executive function in children, adolescents, and older adults. Developmental groups were described broadly; older adults were generally defined as ≥60 years. | Expanded the scope to structured exercise, three executive-function domains, developmental stage, and five exercise-skill characteristics. Non-clinical children, adolescents, and older adults remained eligible, with stage assignment based mainly on study descriptions and age information. | Final title and objectives addressed structured exercise across childhood, adolescence, and later life. Children were defined by mean age <12 years, adolescents by mean age ≥12 to <18 years, and older adults by mean age or lower age limit ≥60 years; explicit rules were added for mixed-age samples. | Clarification and scope refinement. The final review retained the same three developmental populations but replaced broad age descriptions with reproducible thresholds and explicit rules for boundary and mixed-age samples. These refinements affected classification rather than introducing a new population after analysis. |
| 2. Intervention, comparator, and study design | Structured multi-session exercise was framed mainly as open-, closed-, or mixed-skill exercise. Comparators included non-exercise conditions and some active controls such as stretching. Randomized studies were eligible. | Eligible interventions broadened to structured exercise programs that could be coded on five skill characteristics. Concurrent comparators and randomized designs were specified in more detail, including multi-arm, cluster, active-comparator, and crossover trials. | Eligible studies required at least one structured-exercise arm and one concurrent eligible comparator without investigator-prescribed structured exercise. Individually randomized, group/class/cluster-randomized, multi-arm, eligible active nonexercise comparator, and randomized crossover trials were allowed. | Clarification and correction of eligibility rules. Version 3.0 aligned the record with the final screening framework, especially by distinguishing eligible nonexercise comparators from alternative structured-exercise comparators. |
| 3. Executive-function outcome framework | Executive function was defined broadly and included inhibitory control, working memory, cognitive flexibility, and overall executive-function outcomes. A range of behavioral measures was anticipated. | Outcomes were restricted to three predefined domains— inhibitory control, working memory, and cognitive flexibility—using validated neuropsychological or computerized tasks, with the assessment nearest intervention completion selected. | Only performance-based outcomes assignable to one of the three domains were eligible. Rating scales, unassignable global composites, global cognition, academic, fitness, motor, neurophysiological, clinical-symptom, and other non-executive outcomes were excluded. Effects were harmonized so positive Hedges’ g indicated better performance. | Clarification and operational refinement. The final framework standardized outcome-level domain assignment and direction harmonization, determined which outcomes within otherwise eligible studies entered the meta-analysis, and excluded unassignable global executive-function composites from quantitative synthesis. |
| 4. Skill-characteristic coding and reproducibility | Exercise interventions were primarily conceptualized using broad open-, closed-, or mixed-skill categories; detailed arm-level operational definitions and reproducibility procedures were not specified. | A multidimensional arm-level skill-characteristic framework was introduced, including environmental predictability, cognitive engagement, coordination complexity, motor skill learning demand, and broad skill classification; skill progression was additionally recorded. Two-reviewer coding was planned, but the operational decision rules were not yet consolidated into a formal codebook. | Final arm-level consensus coding used environmental predictability, cognitive engagement, coordination complexity, and motor skill learning demand as the principal analyzable dimensions, with broad skill classification and skill progression retained descriptively because of sparse categories. Two reviewers independently coded intervention arms and resolved disagreements by consensus or third-reviewer consultation. The original reviewer-specific pre-consensus coding files were not retained, so formal inter-rater agreement could not be calculated retrospectively. During revision, the operational definitions and decision rules were consolidated and clarified in a revision-stage coding codebook; this did not constitute a new recoding exercise or retrospective reliability assessment. | Methodological clarification and reproducibility enhancement. The multidimensional framework was refined to improve interpretability of heterogeneous exercise interventions. The revision-stage codebook documents the final consensus decision framework but does not retroactively establish original inter-rater reliability. Sparse categories were not forced into inferential models. |
| 5. Search coverage and study identification | Planned sources included Embase, Google Scholar, MEDLINE, PubMed, SSCI, and Scopus. No language or date restrictions were stated. Published and unpublished studies and supplementary identification methods were planned. | Sources were revised to CENTRAL, Embase, PubMed, SCIE, SSCI, and Scopus. Eligibility was limited to English-language reports published from 1 January 2015 to 20 May 2026; only published studies were sought and no supplementary identification methods were used. | The final searches covered PubMed, Embase, the Cochrane Library/CENTRAL, Scopus, and Web of Science from database inception to May 20, 2026, without date or language filters in the database interfaces. During screening, eligibility was restricted to formally published English-language journal articles dated January 1, 2015–May 20, 2026. The 2015 cutoff was a pragmatic scope restriction intended to focus the review on approximately the most recent decade of contemporary randomized evidence; it was not based on an assumption that earlier trials were methodologically inferior. Grey literature, reference-list screening, and forward citation searching were not used. | Post-registration scope restriction and clarification of actual search conduct. The 2015 cutoff, English-language restriction, and journal-publication restriction were based on bibliographic characteristics rather than study findings. These restrictions reduced historical completeness and may have introduced language or publication bias; their implications are discussed explicitly in the manuscript. |
| 6. Data extraction, risk of bias, and certainty | Two reviewers were planned for data extraction and RoB 2 assessment. Study authors were to be contacted for missing information. GRADE was planned for the main outcomes. | Independent extraction and RoB 2 assessment were retained. Author contact remained planned. GRADE was retained but described as to be completed before manuscript submission. | Two reviewers independently completed data extraction and RoB 2 assessment; study authors were not contacted and no individual-participant data were sought. GRADE was completed for overall executive-function performance and the three executive-function domains using GRADEpro GDT. | Correction of actual review conduct and completion of a planned certainty assessment. The decision not to contact study authors represents a departure from the original protocol and may have limited clarification of missing or ambiguous information. |
| 7. Effect-size construction and three-level synthesis | A multilevel random-effects meta-analysis was proposed when multiple dependent effects were available, but effect-size construction and inferential details were broad. | Hedges’ g and a three-level REML model were specified. Change-score effects were preferred when available, otherwise post-intervention group differences were used. Analyses were planned in Stata 18.0. | The primary effect was post-intervention Hedges’ g. A three-level REML model represented sampling variance, residual within-trial heterogeneity, and between-trial heterogeneity. The primary working sampling-variance matrix was diagonal, with Study_ID-clustered CR2 inference and Satterthwaite-adjusted degrees of freedom. Alternative non-diagonal sampling variance–covariance matrices were examined as sensitivity analyses. Actual participant counts were kept distinct from design-adjusted effective sample sizes used for effect-size variance and covariance calculations. Analyses were conducted in R 4.6.1 using metafor 5.0-1 and clubSandwich 0.7.0. | Substantive methodological amendment and reporting clarification. The final framework retained dependent effect sizes while separating random-effects heterogeneity, robust coefficient-level inference, and sampling-covariance assumptions. Distinguishing actual from effective sample sizes improved transparency of participant reporting without altering the variance calculations. |
| 8. Moderators, sensitivity, and reporting bias | Possible separate analyses were anticipated for executive-function domains and broad exercise types; detailed moderator, sensitivity, and reporting-bias procedures were not specified. | Formal models included executive-function domain, developmental stage, five skill characteristics, and a domain × stage interaction. One extreme-value study was to be excluded in sensitivity and small-study-effect analyses; funnel plots and Egger’s test were used. | Executive-function domain and developmental stage were examined in separate three-level meta-regressions. Environmental predictability was treated as the principal skill moderator, cognitive engagement as secondary, coordination complexity as exploratory, and motor skill learning demand as supplementary exploratory; broad skill classification and skill progression were descriptive because of sparse categories. The domain × developmental-stage interaction was not retained because cross-classified cells were too sparse for stable CR2 inference. Sensitivity analyses examined alternative sampling-covariance assumptions, cluster ICCs, change-score construction, baseline imbalance, risk of bias, coding confidence, comparator restrictions, assumed-ICC exclusion, and trial influence. During revision, sampling-covariance sensitivity was extended to six moderator models, and separate developmental-stage covariance and stage-specific leave-one-trial-out analyses were added. The earlier planned exclusion of a study based only on an extreme estimate was abandoned after verification of the source data; no trial was excluded solely because of effect magnitude. Reporting-bias assessment used dependency-aware multilevel diagnostics, GLS study-level aggregation, Egger regression, Kendall rank correlation, and a contour-enhanced funnel plot. | Methodological amendment and robustness expansion. Additional covariance and developmental-stage analyses were introduced during revision to test whether the main conclusions depended on plausible sampling-covariance assumptions or individual trials. These analyses did not change the analytical status of the corresponding moderators and were interpreted as sensitivity analyses rather than new confirmatory hypotheses. Reporting-bias procedures were expanded because conventional asymmetry tests applied directly to all dependent effects would have been inappropriate. |
| 9. Administrative and status corrections | The review was ongoing; the timeline was 22 May 2026 to 10 June 2027. The record selected no funding or institutional support. | The review was marked completed. Beijing Social Science Fund support (23YTC049) was mentioned, but the main funding selection still stated no funding. The timeline remained 22 May 2026 to 10 June 2027. | The final timeline was 20 May 2026 to 26 July 2026 and the review was marked completed. Funding was corrected to Beijing Social Science Fund, grant 23YTC049, with no funder role in design, conduct, analysis, interpretation, writing, or publication decisions. Version 3.0 explicitly stated that it was a retrospective factual update after review completion and did not claim prospective prespecification. | Correction of factual metadata and transparent status reporting. These changes do not alter eligibility or effect estimates, but align the public record with the actual timeline, funding, completion status, and amendment timing. |

Note. PROSPERO registration: CRD420261402803. Version 1.0 was published on May 22, 2026, Version 2.0 on July 14, 2026, and Version 3.0 on July 26, 2026. Versions 2.0 and 3.0 followed the initial synthesis; Version 3.0 was published after completion of the review as a retrospective factual update. Accordingly, decisions introduced or materially refined after the original registration should not be interpreted as prospectively prespecified. This table summarizes changes relevant to eligibility, synthesis, moderator definition, sensitivity analysis, interpretation, reproducibility, or transparency of reporting; minor wording, formatting, and PICO-tag edits are omitted. Revision-stage clarifications, including consolidation of the exercise skill-characteristic coding codebook and additional covariance and developmental-stage robustness analyses, are identified as such and do not retroactively alter the prospective status of the original registration.

**Supplementary Table S6. GRADE certainty-of-evidence assessment for the overall executive-function effect and the three executive-function domains.**

| **Outcome** | **Certainty assessment** | | | | | | **Participants, n (intervention / comparator)** | **Hedges' g (95% CI)** | **Certainty** | **Importance** |
| --- | --- | --- | --- | --- | --- | --- | --- | --- | --- | --- |
|  | **Studies / design** | **Risk of bias** | **Inconsistency** | **Indirectness** | **Imprecision** | **Other considerations** |  |  |  |  |
| Overall executive-function performance | 47 randomized trials | Serious^a^ | Serious^b^ | Not serious | Not serious | Publication bias: not downgradedᶦ | 2199 / 1996 | 0.412 (0.284 to 0.540) | ⊕⊕◯◯ Low^a,b^ | CRITICAL |
| Inhibitory-control performance | 31 randomized trials | Serious^c^ | Serious^d^ | Not serious | Not serious | Publication bias: not downgradedᶦ | 1665 / 1518 | 0.361 (0.174 to 0.548) | ⊕⊕◯◯ Low^c,d^ | CRITICAL |
| Working-memory performance | 22 randomized trials | Serious^e^ | Serious^f^ | Not serious | Not serious | Publication bias: not downgradedᶦ | 1402 / 1301 | 0.351 (0.172 to 0.530) | ⊕⊕◯◯ Low^e,f^ | CRITICAL |
| Cognitive-flexibility performance | 27 randomized trials | Serious^g^ | Serious^h^ | Not serious | Not serious | Publication bias: not downgradedᶦ | 1062 / 973 | 0.521 (0.394 to 0.647) | ⊕⊕◯◯ Low^g,h^ | CRITICAL |

Question: Structured exercise interventions compared with eligible comparator conditions without investigator-prescribed structured exercise for non-clinical children, adolescents, and older adults

Setting: Preschool, school, community, laboratory, sport, fitness, home-based, residential-care, and other health-promotion settings

Explanations

a. Downgraded one level for serious risk of bias because none of the 47 included trials was judged to be at overall low risk of bias; 44 trials had some concerns and three were judged to be at high risk. Concerns were particularly common for selection of the reported result, deviations from intended interventions, and the randomization process. The pooled effect remained stable after excluding the three high-risk trials; therefore, downgrading by two levels was not considered warranted.

b. Downgraded one level for serious inconsistency because substantial unexplained heterogeneity remained across the included evidence (total multilevel *I*² = 74.45%). The 95% prediction interval ranged from −0.476 to 1.299, spanning possible negative or negligible effects through comparatively large positive effects. Although the pooled mean remained positive across the examined sensitivity analyses, substantial variation remained across studies, populations, interventions, and outcome measures; therefore, downgrading by two levels was not considered warranted.

c. Downgraded one level for serious risk of bias because none of the 31 trials contributing inhibitory-control outcomes was judged to be at overall low risk of bias; all 31 trials had some concerns. Concerns were particularly common for selection of the reported result, deviations from intended interventions, and the randomization process. No trial contributing inhibitory-control outcomes was judged to be at overall high risk of bias; therefore, downgrading by two levels was not considered warranted.

d. Downgraded one level for serious inconsistency because substantial unexplained heterogeneity remained among inhibitory-control outcomes (total multilevel I² approximately 79.7%), and the corresponding prediction interval crossed the line of no effect. Although the average pooled estimate was positive, plausible true effects varied substantially across studies, interventions, populations, and inhibitory-control tasks. Downgrading by two levels was not considered warranted.

e. Downgraded one level for serious risk of bias because none of the 22 trials contributing working-memory outcomes was judged to be at overall low risk of bias; 21 trials had some concerns and one trial was judged to be at high risk of bias. Concerns were particularly common for selection of the reported result, deviations from intended interventions, and the randomization process. Because only one contributing trial was at high risk and the remainder had some concerns rather than high risk, downgrading by two levels was not considered warranted.

f. Downgraded one level for serious inconsistency because substantial unexplained heterogeneity remained among working-memory outcomes (total multilevel I² approximately 69.1%), and the corresponding prediction interval crossed the line of no effect. The average pooled estimate was positive, but plausible true effects varied across studies and working-memory measures. Downgrading by two levels was not considered warranted.

g. Downgraded one level for serious risk of bias because none of the 27 trials contributing cognitive-flexibility outcomes was judged to be at overall low risk of bias; 25 trials had some concerns and two trials were judged to be at high risk of bias. Concerns were particularly common for selection of the reported result, deviations from intended interventions, and the randomization process. Because the high-risk trials represented a minority of the contributing evidence, downgrading by two levels was not considered warranted.

h. Downgraded one level for serious inconsistency because important unexplained heterogeneity remained among cognitive-flexibility outcomes (total multilevel I² approximately 61.9%), and the corresponding prediction interval crossed the line of no effect. Although the pooled estimate was positive, plausible true effects varied across studies, populations, interventions, and cognitive-flexibility tasks. Downgrading by two levels was not considered warranted.

i. No additional downgrade was applied for publication bias. Small-study-effect diagnostics were inconsistent: sample-size-based multilevel analyses and aggregated study-level asymmetry tests did not provide statistically significant evidence of small-study effects, whereas standard-error-based multilevel models yielded statistically significant associations that may have been influenced by mathematical coupling. In addition, eligibility was restricted to English-language, formally published journal articles, and grey literature was not searched, reducing the completeness of the evidence base. Publication bias therefore remained uncertain but was not judged sufficiently strongly suspected to warrant an additional GRADE downgrade.

Note. Certainty of evidence was assessed using the GRADE approach. Evidence from randomized controlled trials initially started at high certainty and was considered for downgrading for risk of bias, inconsistency, indirectness, imprecision, and publication bias. Overall executive-function performance and each of the three executive-function domains were rated as low-certainty evidence because of one-level downgrades for serious risk of bias and serious inconsistency. No additional downgrading was applied for indirectness, imprecision, or publication bias. The absence of a publication-bias downgrade should not be interpreted as evidence that publication bias was absent; the relevant statistical diagnostics were inconsistent, and publication-language and publication-status restrictions reduced the completeness of the evidence base. Participant counts are actual analyzed participants and are distinct from design-adjusted effective sample sizes used for effect-size variance calculations. The overall executive-function outcome summarizes the three domains and is not statistically independent of the domain-specific evidence. Because some trials and participant samples contributed outcomes to more than one executive-function domain, domain-specific participant counts overlap and should not be summed. Effect estimates are reported as Hedges’ *g*, with positive values favoring structured exercise. CI, confidence interval; GRADE, Grading of Recommendations Assessment, Development and Evaluation.
